# Innovation in public health surveillance for social distancing during the COVID-19 pandemic: A deep learning and object detection based novel approach

**DOI:** 10.1371/journal.pone.0308460

**Published:** 2024-09-09

**Authors:** Mohammad Arifuzzaman, Rizwan Shaikh, Iftekhar Bhuiyan, S. M. Raiyan Chowdhury, Tauhidul Islam Atoul

**Affiliations:** 1 Dept. of CSE, East West University (EWU), Dhaka, Bangladesh; 2 Dept. of EEE, East West University (EWU), Dhaka, Bangladesh; 3 Dept. of ECE, East West University (EWU), Dhaka, Bangladesh; 4 Dept. of EEE, Bangladesh University of Engineering & Technology (BUET), Dhaka, Bangladesh; UPES Dehradun, INDIA

## Abstract

The Corona Virus Disease (COVID-19) has a huge impact on all of humanity, and people’s disregard for COVID-19 regulations has sped up the disease’s spread. Our study uses a state-of-the-art object detection model like YOLOv4 (You Only Look Once, version 4), a very effective tool, on real-time 25fps, 1920 X 1080 video data streamed live by a camera-mounted Unmanned Aerial Vehicle (UAV) quad-copter to observe proper maintenance of social distance in an area of 35m range in this study. The model has demonstrated remarkable efficacy in identifying and quantifying instances of social distancing, with an accuracy of 82% and little latency. It has been able to work efficiently with real-time streaming at 25–30 ms. Our model is based on CSPDarkNet-53, which was trained on the MS COCO dataset for image classification. It includes additional layers to capture feature maps from different phases. Additionally, the model’s neck is made up of PANet, which is used to aggregate the parameters from various CSPDarkNet-53 layers. The CSPDarkNet-53’s 53 convolutional layers are followed by 53 more layers in the model head, for a total of 106 completely convolutional layers in the design. This architecture is further integrated with YOLOv3, resulting in the YOLOv4 model, which will be used by our detection model. Furthermore, to differentiate humans The aforementioned method was used to evaluate drone footage and count social distance violations in real time. Our findings show that our model was reliable and successful at detecting social distance violations in real-time with an average accuracy of 82%.

## 1 Introduction

By keeping appropriate physical distance, public health officials can prevent the spread of a highly contagious disease. This practice is known as social distancing. Additionally, following the recommendations made by the World Health Organization (WHO), people must keep appropriate social distance from one another to prevent the transmission of the Coronavirus Disease (COVID-19). Depending on the demographics of the area, varying distances may be required to be maintained for safety during a pandemic. Due to the threat posed by SARS-CoV-2, the federal administration, law enforcement, and healthcare ministries have all mandated social distancing measures. To avoid the spread of diseases such as COVID-19, appropriate hygiene and vaccination programs must include social distancing measures. Many efforts have been made and continue to be made to ensure proper social distance between people, but in densely populated areas like South Asia, automated supervision is now necessary to ensure public safety, for which technological platforms such as UAVs and machine learning algorithms can be extremely useful. Scholars in related subjects have undertaken and are still conducting a great deal of study worldwide. Since object detection can be extremely important in this context, numerous models based on neural networks (NN) have been suggested, along with a variety of trainable datasets. For instance, DPMv5-P and DPMv5-C were trained using a fresh dataset that was recently introduced. Their dataset included a sizable collection of object instances that were gathered, annotated, and categorized to develop object identification algorithms [1]. A suggestion to train a deep neural network with a Spatial Pyramid Pooling (SPP) layer was proposed by the authors with the intent of exploring the usefulness and flexibility of solutions that SPP offers [2]. The authors advise training a deep neural network with a Spatial Pyramid Pooling (SPP) layer to investigate the value and adaptability of solutions that SPP provides [3]. On the MS COCO dataset, the Cross Stage Partial Network (CSPNet) has been developed and effectively used [4]. A drone with deep learning capabilities was utilized to identify individuals who were not keeping up their social distance and were exposed [5]. Moreover, the design of an adaptive social distancing detector using YOLOv3 was presented by the authors [6]. The authors investigated the impacts of wind disturbances and high speed on the attitude and altitude control of the STARMAC II quadrotor which was aimed at improving altitude and attitude control of the quadrotor at high speed and in uncontrolled environments [7]. An adaptive social distance detector using YOLOv3, and Gazebo simulation was presented by the authors [8]. A three-axis gimbal system mounted on a mobile platform based on a nonlinear Hammerstein block structure capable of using a Model Predictive Controller(MPC) effectively improving real-time target tracking performance under external disturbances was designed and analyzed by the authors [9]. WilDect-YOLO, an accurate real-time object detection system combining DenseNet, spatial pyramid pooling, and a redesigned path aggregation network, has been proposed for detecting endangered wildlife species across many classes with improved performance by the authors [10]. Moreover, a quick and precise fine-grain object detection model based on the YOLOv4 deep neural network that included Spatial Pyramid Pooling (SPP) and a modified Path Aggregation Network (PANet) proposed by the authors [11]. DenseSPH-YOLOv5, a real-time DL-based high-performance damage detection model, in which DenseNet blocks have been merged with the backbone was designed by the authors. Their model was further improved by Convolutional Block Attention Modules (CBAM) producing superior detection capabilities owing to robust and discriminating deep spatial feature extraction [12]. There have been several related research works off late [13–18]. However, in this study, we applied the YOLOv4 algorithm on a real-time video dataset broadcast by a camera placed on a UAV quadcopter, which calculates the distance between people by taking into account both their 2-D co-ordinates from bounding box regression and the depth of the object.

To produce expected results, we had to calibrate our camera to estimate distances among objects. We implemented this by using photos of a meter scale taken from different angles. Furthermore, key criteria in our analysis include confidence, non-maximum suppression threshold, minimum pixel distance, known distance, and known width. To improve performance in low-light conditions, a confidence level of 0.3 (on a scale of 0 to 1) was set up whereas, the non-maximum suppression threshold was chosen to be 0.4. A reference image with a known distance of 1.2 meters was used for further calibration of our model.

With the aid of the cutting-edge YOLOv4 model and appropriate geometrical analysis, our study examines a feasible method for identifying social distance violations and measuring distances in real-time. Because the study was done in real-time, the results are constrained by the lack of ground truth data.

We developed a novel approach for detecting social distancing violations during the COVID-19 pandemic, which applies to any situation that requires adherence to social distancing norms. Despite using an existing object detection technique, our approach sets our research apart.

The significant contributions of our research work are listed below.

- Introducing a novel UAV-based surveillance method for preserving public health protocol i.e. maintaining social distancing
- Proposing an effective model for detecting social distance violations in public spaces with high accuracy
- Implementing a mathematical approach based on Euclidean geometry in conjunction with a memory-efficient YOLOv4 object identification model to achieve high accuracy in measuring distances between objects in a video captured from a UAV

The paper is structured as follows: Section 3 discusses the hardware design, Section 4 describes the data, Section 5 elaborates on the design of the detection model, Section 6 provides a summary of the results followed by the conclusion section.

## 2 Methodology

**Fig 1:**
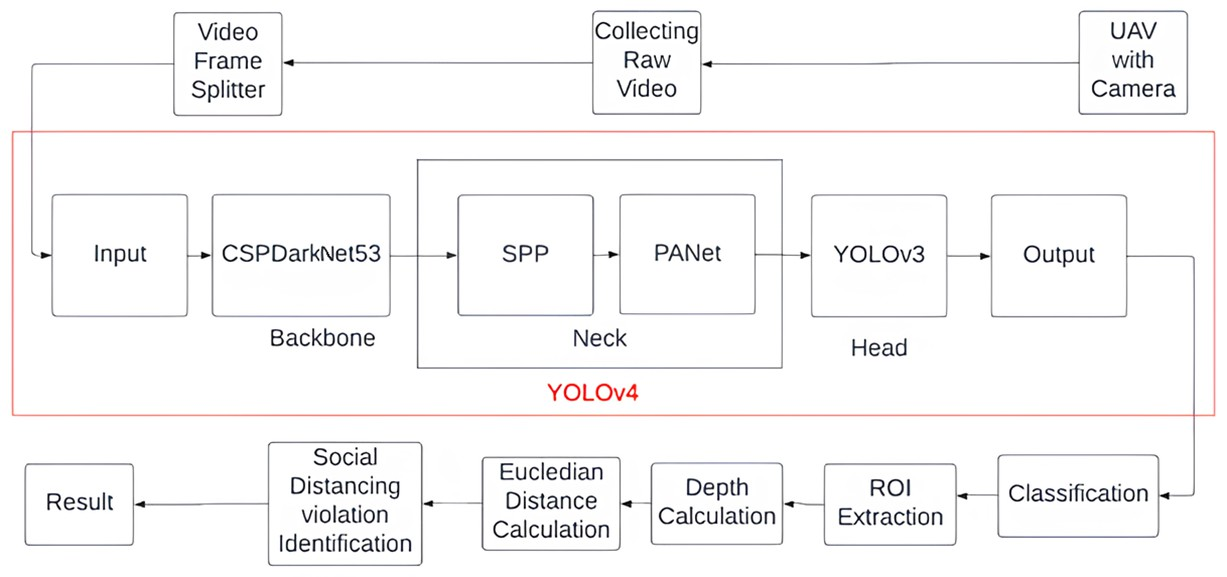
Block diagram of the proposed model.

Our research can be categorized into two parts-Hardware and Software (Detection). The hardware part comprises a camera-mounted UAV quad-copter which has been used to collect real-time videos for feeding into the software model which performs the detection of social distance violations using YOLOv4 architecture. We have obtained prior written approval for human subjects research from a locally authorized entity (Banasree Society) which is equivalent to an Institutional Review Board (IRB) or equivalent ethics committee as per the requirements of the journal. The summarized methodology has been illustrated in the following diagram (Fig 1).

The key assumptions underlying our model are:

- Good weather condition with high visibility(greater than 70m)
- Moderate wind speed(8-12 mph)
- Moderate precipitation ensuring efficient camera functionality

## 3 Hardware implementation

In our work, we used a UAV (Unmanned Aerial Vehicle) quad-copter to capture videos to detect violations of social distancing in real time. For analyzing the motion of the quad-copter in 3-D space, we must consider the kinematics, torque, rotation matrix, and the two coordinate systems [19]. One of them is the Body coordinate system which is indexed ‘*b*’ and is affected by the motion of the UAV whereas the Ground coordinate system which is indexed ‘n’ deals with the influence of gravitational forces. The Body coordinate system travels with the quad-copter, albeit the ground coordinate system is taken as the fixed reference for the quad-copter. They are described below in brief.

### 3.1 The kinematics

The kinematics involves studying the motion of the system of bodies without forces or potential fields affecting the motion. It shows how momentum and energy are shared among interacting bodies. It is essential for the guidance and navigation of the drone alongside the design of the control system for maneuvering it.

### 3.2 Torque

A torque indicates the amount of force that may rotate a drone around an axis. Just as force is utilized to identify what causes the drone to accelerate in linear kinematics, torque must be investigated to ascertain what causes the drone to gain rotational acceleration.

### 3.3 Rotational matrix

The rotating matrix allows a drone to be moved as a rigid unit without affecting its internal geometry. It is defined as a 3 X 3 matrix that modifies the vector’s magnitude but not direction when multiplied by a vector.

### 3.4 Ground coordinate system

**Fig 2:**
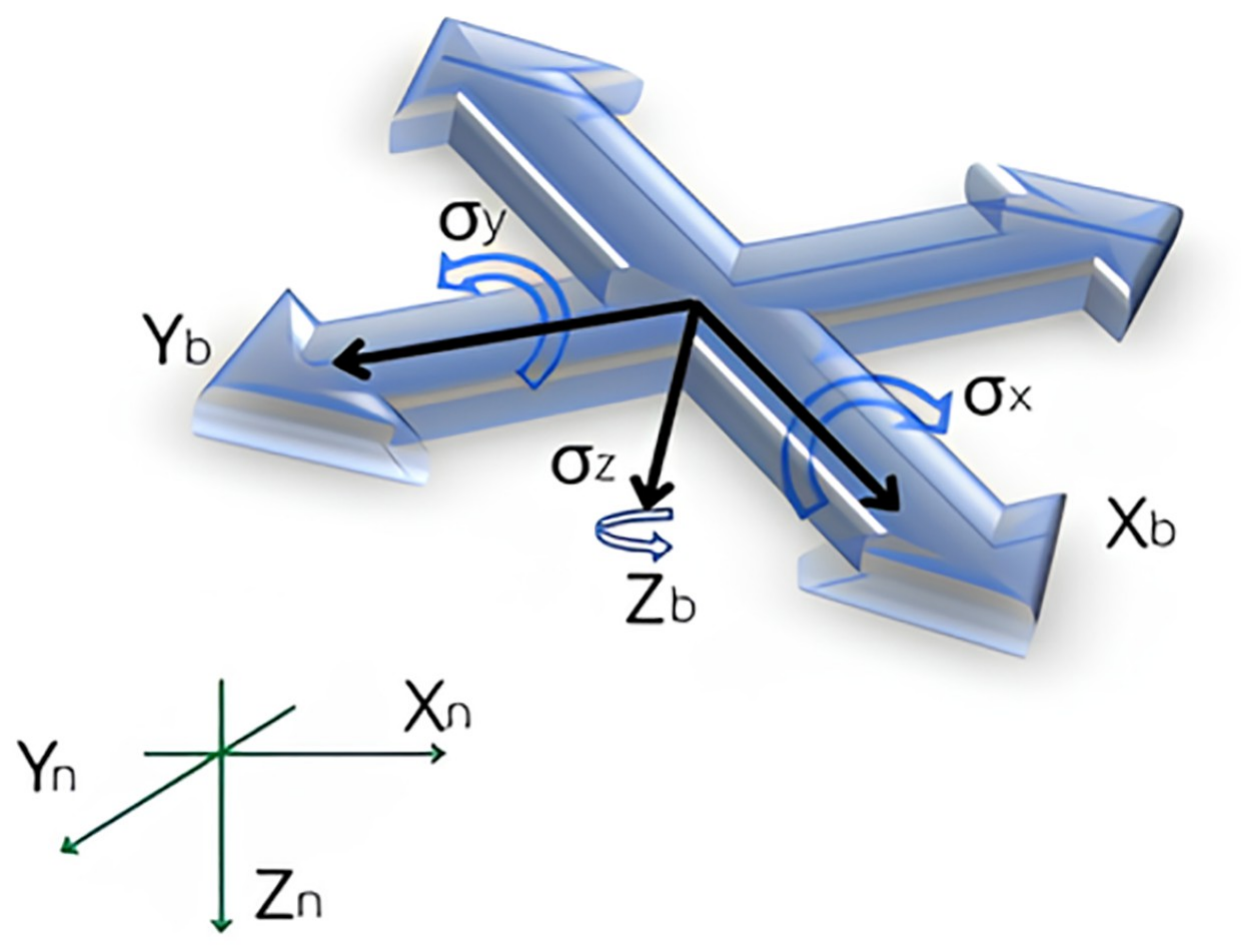
The ground and body coordinate systems.

The Ground coordinate system is mainly used to describe the motion of the quadcopter relative to the ground and to obtain its position. The ground coordinate system, taken as the reference, is fixed on the ground level of the observation area where the origin can be chosen to be any point on the ground. Typically, the take-off point of the quadcopter is assumed to be the origin of this system. The three axes of this system follow the right-hand rule. (Fig 2) Right-hand rule indicates the direction of the coordinate axes. When a person holds the thumb, index finger, and middle finger of his/her right hand so that they form three right angles, then the thumb symbolizes the x-axis, the index finger the y-axis, and the middle finger the z-axis.

### 3.5 Body coordinate system

**Fig 3:**
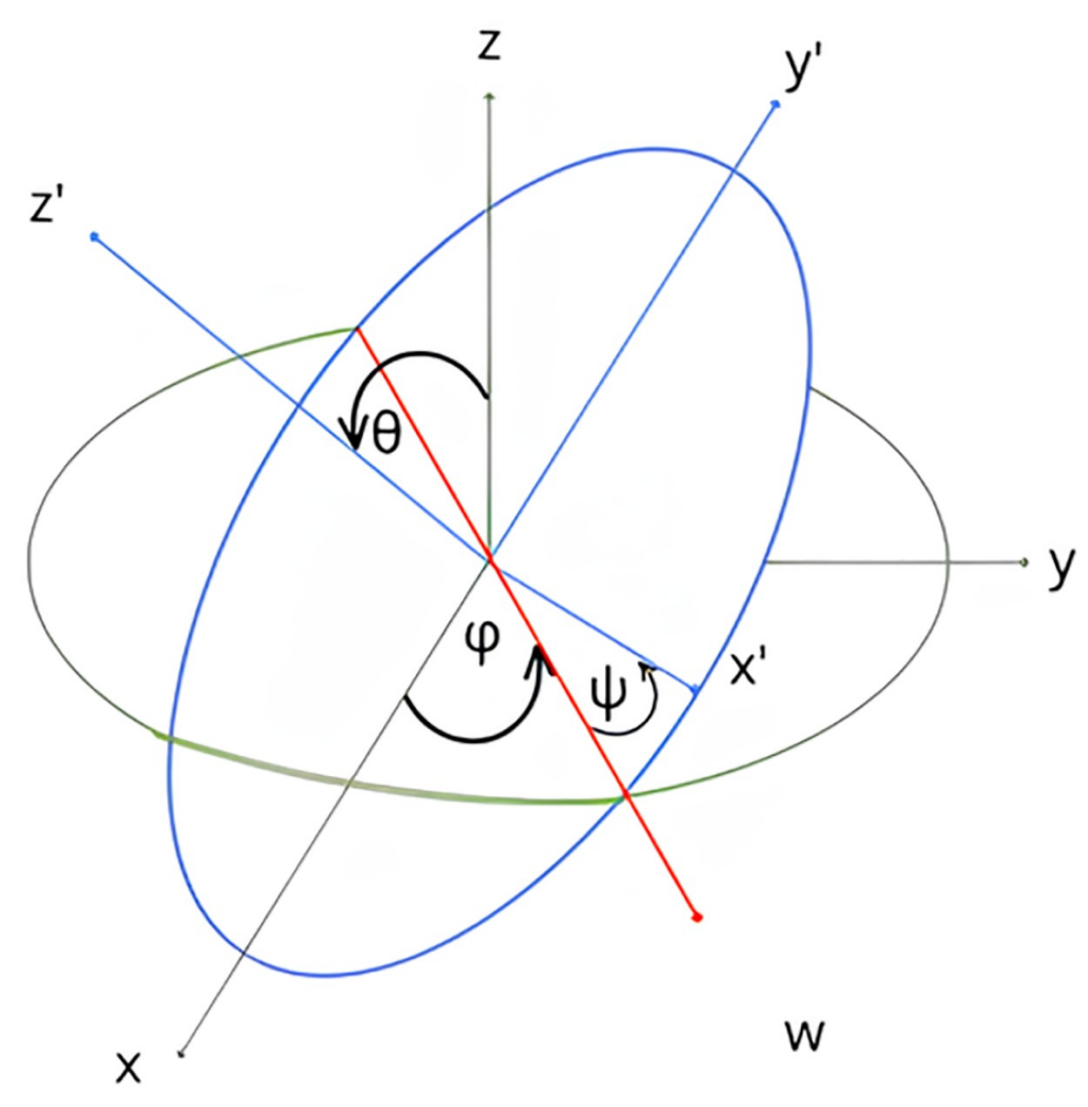
Euler angles.

The origin of the Body coordinate system is located at the center of gravity of the device that is moving with respect to the Ground coordinate system. To obtain the orientation of the quadcopter, the Body coordinate system must be linked to the Ground coordinates. Euler angles are utilized to establish this relation. The three Euler angles are Φ, *θ*, Ψ and they are known as roll, pitch, and yaw respectively where roll is the term for rotation about the front-to-back axis, pitch is the term for rotation about the side-to-side axis, and yaw is the name for rotation about the vertical axis (Fig 3).

The Euler angles dictate the transformation relation of the coordinate system as shown in the transformation matrices given below [20].

$$\begin{array}{cc} & R\left(x,\Phi \right)=[\begin{array}{ccc} 1 & 0 & 0\\ 0 & \text{cos}\;\Phi & -\text{sin}\;\Phi \\ 0 & \text{sin}\;\Phi & \text{cos}\;\Phi \end{array}]\\ & R\left(y,\theta \right)=[\begin{array}{ccc} \text{cos}\;\theta & 0 & \text{sin}\;\theta \\ 0 & 1 & 0\\ -\text{sin}\;\theta & 0 & \text{cos}\;\theta \end{array}]\\ & R\left(z,\Psi \right)=[\begin{array}{ccc} \text{cos}\;\Psi & -\text{sin}\;\Psi & 0\\ \text{sin}\;\Psi & \text{cos}\;\Psi & 0\\ 0 & 0 & 1 \end{array}] \end{array}$$ (1)

Here, **R**(*x*, Ψ), *R*(*y*, *θ*), *R*(*x*, Φ) are the transformation matrices.

The transfer matrix, **R** = *R*(*z*, Ψ)*R*(*y*, *θ*)*R*(*x*, Φ) is given below.

$$\begin{array}{cc} & R=[\begin{array}{ccc} \text{cos}\;\Psi \;\text{cos}\;\theta & \text{cos}\;\Psi \;\text{sin}\;\theta \;\text{sin}\;\Phi & \text{cos}\;\Psi \;\text{sin}\;\theta \;\text{cos}\;\Phi \\ & -\text{sin}\;\Psi \;\text{cos}\;\Phi & +\text{sin}\;\Psi \;\text{sin}\;\Phi \\ \text{sin}\;\Psi \;\text{cos}\;\theta & \text{sin}\;\Psi \;\text{sin}\;\theta \;\text{sin}\;\Phi & \text{sin}\;\Psi \;\text{sin}\;\theta \;\text{cos}\;\Phi \\ & +\text{cos}\;\Psi \;\text{cos}\;\Phi & -\text{cos}\;\Psi \;\text{sin}\;\Phi \\ -\text{sin}\;\theta & \text{cos}\;\theta \;\text{sin}\;\Phi & \text{cos}\;\theta \;\text{cos}\;\Phi \end{array}] \end{array}$$ (2)

Let us define *p*, *q*, *r* as the angular velocity of the three axes in the Body coordinate system and Ψ, *θ*, Ψ are the Euler angular velocities in the Ground coordinate system, The relationship between the Euler angular velocity and the Body angular velocity is given by the following equations.

$$\begin{array}{cc} \left[\begin{array}{c} p\\ q\\ r \end{array}\right] & =[\begin{array}{ccc} 1 & 0 & -\text{sin}\;\theta \\ 0 & \text{cos}\;\Phi & \text{sin}\;\Phi \;\text{cos}\;\theta \\ 0 & -\text{sin}\;\Phi & \text{cos}\;\Phi \;\text{cos}\;\theta \end{array}][\begin{array}{c} \dot{\Phi }\\ \dot{\theta }\\ \dot{\Psi } \end{array}]\\ & =[\begin{array}{c} \dot{\Phi }-\dot{\Psi }\text{sin}\;\theta \\ \dot{\theta }\;\text{cos}\;\Phi +\dot{\Psi }\text{sin}\;\Phi \;\text{cos}\;\theta \\ -\dot{\theta }\;\text{sin}\;\Phi +\dot{\Psi }\text{cos}\;\Phi \;\text{cos}\;\theta \end{array}] \end{array}$$ (3)

The equation given above can also be transformed as:

$$\begin{array}{cc} \left[\begin{array}{c} \dot{\Phi }\\ \dot{\theta }\\ \dot{\Psi } \end{array}\right] & =[\begin{array}{ccc} 1 & \text{sin}\;\Phi \;\text{tan}\;\theta & \text{cos}\;\phi \;\text{tan}\;\theta \\ 0 & \text{cos}\;\Phi & -\text{sin}\;\Phi \\ 0 & \text{sin}\;\Phi \;\text{sec}\;\theta & \text{cos}\;\Phi \;\text{sec}\;\theta \end{array}][\begin{array}{c} p\\ q\\ r \end{array}]\\ & =[\begin{array}{c} p+q\;\text{sin}\;\Phi \;\text{tan}\;\theta +r\;\text{cos}\;\Phi \;\text{tan}\;\theta \\ q\;\text{cos}\;\Phi -r\;\text{sin}\;\Phi \\ (q\;\text{sin}\;\Phi +r\;\text{cos}\;\Phi )/\text{cos}\;\theta \end{array}] \end{array}$$ (4)

This can be reduced to a standard unit matrix for a stabilized flight of the quadcopter.

$$\begin{array}{cc} \left[\begin{array}{c} \dot{\Phi }\\ \dot{\theta }\\ \dot{\Psi } \end{array}\right] & =[\begin{array}{ccc} 1 & 0 & 0\\ 0 & 1 & 0\\ 0 & 0 & 1 \end{array}][\begin{array}{c} p\\ q\\ r \end{array}] \end{array}$$ (5)

To design a dynamic model, determining the forces and torques acting on the quadcopter is imperative. The forces must also be organized in such a way so that their influence on the quadcopter can be measured precisely irrespective of the orientation of the quadcopter. The vector of the orientation of the quadcopter must be rotated to determine how much force the motors must put out to keep it hovering by overcoming gravitational force [21]. The rotational matrices applicable to rotate about a single axis are given below.

$$\begin{array}{cc} & R_{\sigma x}\left(\Phi \right)=[\begin{array}{ccc} 0 & \text{cos}\;\Phi & \text{sin}\;\Phi \\ 1 & 0 & 0\\ 0 & -\text{sin}\;\Phi & \text{cos}\;\Phi \end{array}]\\ & R_{\sigma y}\left(\theta \right)=[\begin{array}{ccc} 0 & 1 & 0\\ \text{cos}\;\theta & 0 & \text{sin}\;\theta \\ \text{sin}\;\theta & 0 & \text{cos}\;\theta \end{array}]\\ & R_{\sigma z}\left(\Psi \right)=[\begin{array}{ccc} \text{sin}\;\Psi & \text{cos}\;\Psi & 0\\ \text{cos}\;\Psi & \text{sin}\;\Psi & 0\\ 0 & 0 & 1 \end{array}] \end{array}$$ (6)

To calculate the total conversion from the Body frame to the Ground frame, all three matrices can be multiplied together to obtain the complete rotation matrix.

$$\begin{array}{c} C_{b}^{n}=R_{\sigma x}\left(\Phi \right)R_{\sigma y}\left(\theta \right)R_{\sigma z}\left(\Psi \right) \end{array}$$ (7)

The transformation from the Body to the Ground frame is implemented by the computed matrix. In certain situations, calculating the conversion from the Ground to the Body frame is also necessary. To accomplish this, transpose the outcome to get $C_{n}^{b}$ matrix.

$$\begin{array}{c} C_{n}^{b}={\left[C_{b}^{n}\right]}^{T} \end{array}$$ (8)

**Fig 4:**
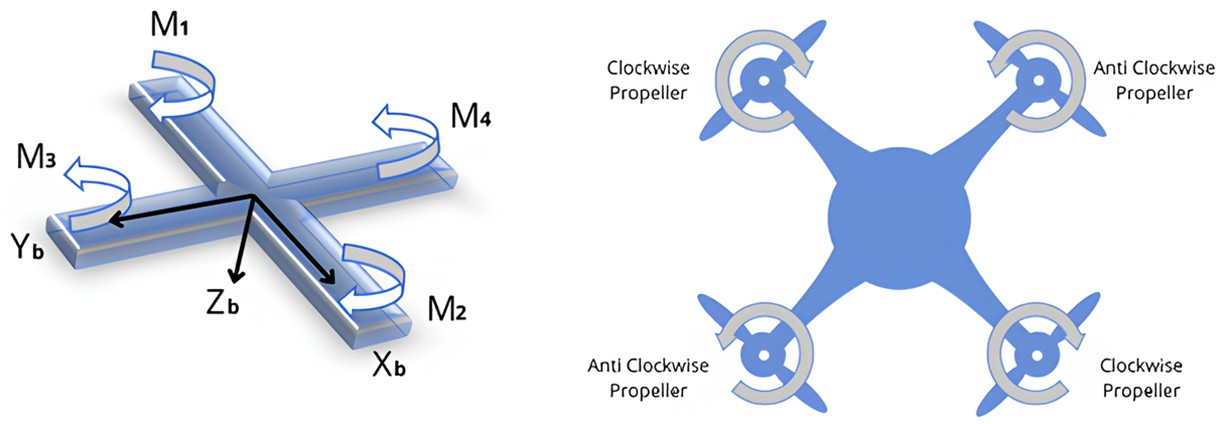
Directions of rotation of the motors.

The alignment of the motors plays a vital role in maintaining the stability of the quadcopter. Motor one and two having a CW(Clockwise) rotation are installed parallel to the x-axis, whereas the other two CCW(Counterclockwise) motors are mounted on the y-axis (Fig 4).

To properly model and incorporate the aerodynamic effects on the UAV, the drag force generated by the air resistance is included too in the following manner.

$$\begin{array}{cc} \left[\begin{array}{c} \ddot{x}\\ \ddot{y}\\ \ddot{z} \end{array}\right] & =-g[\begin{array}{c} 0\\ 0\\ 1 \end{array}]-\frac{1}{m}[\begin{array}{ccc} A_{x} & 0 & 0\\ 0 & A_{y} & 0\\ 0 & 0 & A_{z} \end{array}][\begin{array}{c} \dot{x}\\ \dot{y}\\ \dot{z} \end{array}]\\ & +\frac{T}{m}[\begin{array}{c} \text{cos}\;\Phi \;\text{sin}\;\theta \;\text{cos}\;\Psi +\text{sin}\;\Phi \;\text{sin}\;\Psi \\ \text{sin}\;\Phi \;\text{sin}\;\theta \;\text{cos}\;\Psi -\text{cos}\;\Phi \;\text{sin}\;\Psi \\ \text{cos}\;\theta \;\text{cos}\;\Psi \end{array}] \end{array}$$ (9)

Where, *A*_*x*_, *A*_*y*_, *A*_*z*_ are the drag force coefficients for velocities in the corresponding directions of the inertial frame. For formalizing the kinematics, let us define the dynamic state of the quadcopter w.r.t the inertial frame as **x** = [*x*, *y*, *z*]^*T*^ and $\dot{x}={\left[\dot{x},\dot{y},\dot{z}\right]}^{T}$, respectively. Similarly, we define the roll, pitch, and yaw angles in the body frame as *θ* = [Φ, *θ*, Ψ]^*T*^ with corresponding angular velocities $\dot{\theta }={\left[\dot{\Phi },\dot{\theta },\dot{\Psi }\right]}^{T}$. We can relate the body and inertial frame by a rotation matrix *R* as given below.

$$\begin{array}{c} R=[\begin{array}{ccc} \text{cos}\;\Phi \;\text{cos}\;\Psi & -\text{cos}\;\Psi \;\text{sin}\;\Phi & \text{sin}\;\theta \;\text{sin}\;\Psi \\ -\text{cos}\;\theta \;\text{sin}\;\Phi \;\text{sin}\;\Psi & -\text{cos}\;\Phi \;\text{cos}\;\theta \;\text{sin}\;\Psi & \\ \text{cos}\;\theta \;\text{cos}\;\Psi \;\text{sin}\;\Phi & \text{cos}\;\Phi \;\text{cos}\;\theta \;\text{cos}\;\Psi & -\text{cos}\;\Psi \;\text{sin}\;\theta \\ +\text{cos}\;\Phi \;\text{sin}\;\Phi & -\text{sin}\;\Phi \;\text{sin}\;\Psi & \\ \text{sin}\;\Phi \;\text{sin}\;\theta & \text{cos}\;\Phi \;\text{sin}\;\theta & \text{cos}\;\theta \end{array}] \end{array}$$ (10)

The state variables of the quadrotor are the following twelve quantities:

- (*x*, *y*, *z*)—Position of the center of mass of the quadrotor in the Inertial frame of reference.
- (*θ*, Φ, Ψ)—Rotation of Euler angles from Inertial to Body frame of reference.
- (*v*_*bx*_, *v*_*by*_, *v*_*bz*_)—Linear velocities measured along each axis in the Body frame of reference.
- (*ω*_*bx*_, *ω*_*by*_, *ω*_*bz*_)—Angular velocities measured along each axis in the Body frame of reference.

The states (*x*, *y*, *z*) are Inertial (Ground) frame quantities, whereas (*v*_*bx*_, *v*_*by*_, *v*_*bz*_) are the Body frame quantities. However, assuming the rotation applied from Inertial to Body follows sequence 1-2-3, there is a relationship between angular position and velocities.

$$\begin{array}{cc} & \frac{d}{dt}\left[\begin{array}{c} x\\ y\\ z \end{array}\right]=R_{b}^{i}v_{b}={\left[R_{i}^{b}\right]}^{T}[\begin{array}{c} v_{bx}\\ v_{by}\\ v_{bz} \end{array}]=\\ & \left[\begin{array}{ccc} \text{cos}\;\Psi \;\text{cos}\;\theta & \text{sin}\;\theta \;\text{sin}\;\Psi \;\text{cos}\;\Psi & \text{sin}\;\Psi \;\text{sin}\;\theta \\ & -\text{sin}\;\Psi \;\text{cos}\;\Phi & +\text{sin}\;\theta \;\text{cos}\;\Psi \;\text{cos}\;\Phi \\ \text{sin}\;\Phi \;\text{cos}\;\theta & \text{sin}\;\theta \;\text{sin}\;\Phi \;\text{sin}\;\Phi & \text{cos}\;\Phi \;\text{sin}\;\theta \;\text{sin}\;\Phi \\ & +\text{cos}\;\theta \;\text{cos}\;\Phi & -\text{sin}\;\Phi \;\text{cos}\;\Psi \\ -\text{sin}\;\theta & \text{sin}\;\theta & \text{cos}\;\theta \;\text{cos}\;\Phi \end{array}][\begin{array}{c} v_{bx}\\ v_{by}\\ v_{bz} \end{array}\right] \end{array}$$ (11)

We can set the following identity matrix for relating Euler angles to angular velocities.

$$\begin{array}{c} R_{\text{Roll}}^{b}\left(\Phi \right)=R_{\text{Yaw}}^{\text{Roll}}\left(\Psi \right)=R_{\text{Pitch}}^{\text{Roll}}\left(\theta \right)=I \end{array}$$ (12)

Then,

$$\begin{array}{cc} \left[\begin{array}{c} \omega_{bx}\\ \omega_{by}\\ \omega_{bz} \end{array}\right] & =R_{\text{Roll}}^{b}\left(\Phi \right)[\begin{array}{c} \Phi \\ 0\\ 0 \end{array}]+R_{\text{Roll}}^{b}\left(\Phi \right)R_{\text{Pitch}}^{\text{Roll}}\left(\theta \right)[\begin{array}{c} 0\\ \theta \\ 0 \end{array}]\\ & +R_{\text{Roll}}^{b}\left(\Phi \right)R_{\text{Yaw}}^{\text{Roll}}\left(\Psi \right)R_{\text{Pitch}}^{\text{Roll}}\left(\theta \right)[\begin{array}{c} 0\\ 0\\ \Psi \end{array}] \end{array}$$ (13)

We can write the above equation in a more compact form as shown below.

$$\begin{array}{cc} \left[\begin{array}{c} \omega_{bx}\\ \omega_{by}\\ \omega_{bz} \end{array}\right] & =[\begin{array}{c} \Phi \\ 0\\ 0 \end{array}]+R_{\text{Roll}}^{b}\left(\Phi \right)[\begin{array}{c} 0\\ \theta \\ 0 \end{array}]+R_{\text{Roll}}^{b}\left(\Phi \right)R_{\text{Pitch}}^{\text{Roll}}\left(\theta \right)[\begin{array}{c} 0\\ 0\\ \Psi \end{array}]\\ & =[\begin{array}{c} \Phi \\ 0\\ 0 \end{array}]+[\begin{array}{ccc} 1 & 0 & 0\\ 0 & \text{cos}\;\Phi & \text{sin}\;\Phi \\ 0 & -\text{sin}\;\Phi & \text{cos}\;\Phi \end{array}][\begin{array}{c} 0\\ \theta \\ 0 \end{array}]\\ & +[\begin{array}{ccc} 1 & 0 & 0\\ 0 & \text{cos}\;\Phi & \text{sin}\;\Phi \\ 0 & -\text{sin}\;\Phi & \text{cos}\;\Phi \end{array}][\begin{array}{ccc} \text{cos}\;\theta & 0 & \text{cos}\;\theta \\ 0 & 1 & 0\\ \text{cos}\;\theta & 0 & \text{cos}\;\theta \end{array}][\begin{array}{c} 0\\ 0\\ \Psi \end{array}]\\ & =[\begin{array}{ccc} 1 & 0 & -\text{sin}\;\theta \\ 0 & \text{cos}\;\Phi & \text{sin}\;\Phi \;\text{cos}\;\theta \\ 0 & -\text{sin}\;\theta & \text{cos}\;\Phi \;\text{cos}\;\theta \end{array}][\begin{array}{c} \Phi \\ \theta \\ \Psi \end{array}] \end{array}$$ (14)

Inverting the coefficient transformation matrix, we get the following relation.

$$\begin{array}{c} \left[\begin{array}{c} \Phi \\ \theta \\ \Psi \end{array}]=[\begin{array}{ccc} 1 & \text{sin}\;\Phi \;\text{tan}\;\theta & \text{cos}\;\Phi \;\text{tan}\;\theta \\ 0 & \text{cos}\;\Phi & \text{sin}\;\Phi \;\text{cos}\;\theta \\ 0 & \text{sin}\;\Phi \;\text{sec}\;\theta & \text{cos}\;\Phi \;\text{sec}\;\theta \end{array}][\begin{array}{c} \omega_{bx}\\ \omega_{by}\\ \omega_{bz} \end{array}\right] \end{array}$$ (15)

For analysis of the kinetic model of the quadcopter, the following equations were used.

$$\begin{array}{cc} & m\dot{v}=F_{f}+F_{d}+F_{g} \end{array}$$ (16)

$$\begin{array}{cc} & J\dot{\Omega }+\Omega \times J\Omega =M_{f}-M_{d}+M_{c} \end{array}$$ (17)

Here, *M*_*c*_ is the Coriolis torque which can be written as shown below.

$$\begin{array}{c} M_{c}=\sum_{i=1}^{i=4}\Omega \times J_{r}[\begin{array}{c} 0\\ 0\\ {\left(-1\right)}^{i+1}\omega_{i} \end{array}] \end{array}$$ (18)

Here, *J*_*r*_ = Moment of inertia of the rotor. Assuming *J*_*r*_ is very negligible, the above equations can be simplified as follows.

$$\begin{array}{cc} & m\dot{v}=F_{f}+F_{g} \end{array}$$ (19)

$$\begin{array}{cc} & J\dot{\Omega }+\Omega \times J\Omega =M_{f} \end{array}$$ (20)

Here, *M*_*f*_ is a vector containing the specific roll, pitch, and yaw torques.

$$\begin{array}{cc} F_{f} & =R[\begin{array}{c} 0\\ 0\\ \sum_{i=1}^{i=4}\;F_{i} \end{array}]\\ & =[\begin{array}{c} \text{cos}\;\Phi \;\text{cos}\;\Psi \;\text{sin}\;\theta +\text{sin}\;\Phi \;\text{sin}\;\Psi \\ \text{cos}\;\Phi \;\text{sin}\;\theta \;\text{sin}\;\Psi -\text{cos}\;\Psi \;\text{sin}\;\Phi \\ \text{cos}\;\theta \;\text{cos}\;\Phi \end{array}]\sum_{i=1}^{i=4}F_{i} \end{array}$$ (21)

The lift produced by the i-th motor can be expressed as shown below.

$$\begin{array}{c} F_{i}=b\omega_{i}^{2}\;;\;i=1,2,3,4 \end{array}$$ (22)

Here, b is the lift coefficient of the motors. The gravitational force on the quadcopter can be written as shown below.

$$\begin{array}{c} F_{g}=[\begin{array}{c} 0\\ 0\\ -mg \end{array}] \end{array}$$ (23)

Hence, the accelerations along the three axes can be determined from the equation shown below.

$$\begin{array}{c} \left[\begin{array}{c} \ddot{x}\\ \ddot{y}\\ \ddot{z} \end{array}]=\frac{b}{m}[\begin{array}{c} \text{cos}\;\Phi \;\text{cos}\;\Psi \;\text{sin}\;\theta +\text{sin}\;\Phi \;\text{sin}\;\Psi \\ \text{cos}\;\Phi \;\text{sin}\;\theta \;\text{sin}\;\Psi -\text{cos}\;\Psi \;\text{sin}\;\Phi \\ \text{cos}\;\theta \;\text{cos}\;\Phi \end{array}]\sum_{i=1}^{i=4}\omega_{i}^{2}-[\begin{array}{c} 0\\ 0\\ g \end{array}\right] \end{array}$$ (24)

The gyro moment of the quadcopter can be determined from the following equation.

$$\begin{array}{c} \Omega \times J\Omega =[\begin{array}{c} p\\ q\\ r \end{array}]\times [\begin{array}{c} pl_{x}\\ ql_{y}\\ rl_{z} \end{array}]=[\begin{array}{c} qr(l_{z}-l_{y})\\ pr(l_{x}-l_{z})\\ pq(l_{y}-l_{x}) \end{array}] \end{array}$$ (25)

The specific roll, pitch and yaw torques can be determined from the following equation.

$$\begin{array}{c} M_{f}=[\begin{array}{c} qr(l_{z}-l_{y})\\ pr(l_{x}-l_{z})\\ pq(l_{y}-l_{x}) \end{array}]+[\begin{array}{c} Lb(\omega_{4}^{2}-\omega_{2}^{2})\\ Lb(\omega_{3}^{2}-\omega_{1}^{2})\\ d(\omega_{1}^{2}+\omega_{3}^{2}-\omega_{2}^{2}-\omega_{4}^{2}) \end{array}] \end{array}$$ (26)

Hence, the corresponding angular accelerations can be written as given below.

$$\begin{array}{c} \left[\begin{array}{c} \dot{p}\\ \dot{q}\\ \dot{r} \end{array}]=[\begin{array}{c} qr\left(l_{z}-l_{y}\right)/l_{x}\\ pr\left(l_{x}-l_{z}\right)/l_{y}\\ pq\left(l_{y}-l_{x}\right)/l_{z} \end{array}]+[\begin{array}{c} Lb\left(\omega_{4}^{2}-\omega_{2}^{2}\right)/l_{x}\\ Lb\left(\omega_{3}^{2}-\omega_{1}^{2}\right)/l_{y}\\ d\left(\omega_{1}^{2}+\omega_{3}^{2}-\omega_{2}^{2}-\omega_{4}^{2}\right)/l_{z} \end{array}\right] \end{array}$$ (27)

Brushless motors have been used for the design of our quadcopter. For our electric motors, the produced torque is given by the following equation.

$$\begin{array}{c} \tau =K_{t}\left(I-I_{0}\right) \end{array}$$ (28)

Where, *τ* is the Torque developed inside the motor, *I* is the current input to the motor, *I*_0_ is the no-load current, *K*_*t*_ is the torque constant of the motor. The voltage across the motor is the sum of the back-EMF and resistive loss.

$$\begin{array}{c} V=IR_{m}+K_{v}\omega \end{array}$$ (29)

Where, *V* is the Voltage drop across the motor, *R*_*m*_ is the motor resistance, *ω* is the angular velocity, *K*_*v*_ is the motor constant proportional to the back-emf generated per rpm. We can use this description of our motor to calculate the power it consumes.

$$\begin{array}{c} P=IV=\frac{\left(\tau +K_{t}I_{0}\right)\left(K_{t}I_{0}R_{m}+\tau R_{m}+\omega K_{t}K_{v}\right)}{K_{t}^{2}} \end{array}$$ (30)

Assuming negligible motor resistance and *K*_*t*_*I*_0_ << *τ*, we can simplify this equation as follows.

$$\begin{array}{c} P\approx \frac{\tau K_{v}\omega }{K_{t}} \end{array}$$ (31)

A complex system of forces and torques dictates the motion of the quadcopter. The governing equations of the system of forces are given below [22].

$$\begin{array}{c} P=\frac{T^{3/2}}{\sqrt{2\rho A}} \end{array}$$ (32)

$$\begin{array}{c} T_{B}=\sum_{i=1}^{4}T_{i}=[\begin{array}{c} 0\\ 0\\ b\sum \omega_{i}^{2} \end{array}] \end{array}$$ (33)

$$\begin{array}{c} F_{D}=-k_{d}[\begin{array}{c} \dot{x}\\ \dot{y}\\ \dot{z} \end{array}] \end{array}$$ (34)

Where, *P* is the power consumed by the motor, *T* is the thrust produced by the motor, *ρ* is the density of air, *A* is the swept area of the rotor, *T*_*B*_ is the total thrust generated by the motors, *F*_*D*_ is the drag force acting on the quad-copter. We can determine the complete torque about the z-axis for the i-th motor as shown in the equation below [23].

$$\begin{array}{c} \tau_{z}=d\omega^{2}+I_{M}\dot{\omega } \end{array}$$ (35)

Here, $\dot{\omega }$ and *ω* are the angular acceleration and angular velocity of the motor. For a steady flight, we have $\dot{\omega }=0$. We can write the overall torque about the z-axis as shown below.

$$\begin{array}{c} \tau_{\Psi }=d\left(\omega_{1}^{2}-\omega_{2}^{2}+\omega_{3}^{2}-\omega_{4}^{2}\right) \end{array}$$ (36)

The roll and pitch torques are derived from standard mechanics. The roll torque is given by the following equation.

$$\begin{array}{c} \tau_{\Phi }=\sum r\times T=Lb\left(\omega_{3}^{2}-\omega_{1}^{2}\right) \end{array}$$ (37)

Similarly,

$$\begin{array}{c} \tau_{\theta }=\sum r\times T=Lb\left(\omega_{4}^{2}-\omega_{2}^{2}\right) \end{array}$$ (38)

We can represent the overall torque produced by the motors as a matrix as shown below.

$$\begin{array}{c} \tau_{B}=[\begin{array}{c} Lb(\omega_{4}^{2}-\omega_{2}^{2})\\ Lb(\omega_{3}^{2}-\omega_{1}^{2})\\ d(\omega_{1}^{2}-\omega_{2}^{2}+\omega_{3}^{2}-\omega_{4}^{2}) \end{array}] \end{array}$$ (39)

Where, *d* is the drag coefficient and *L* is the distance from the center of the quad-copter to any of the propellers.

### 3.6 Quadcopter design

**Fig 5:**
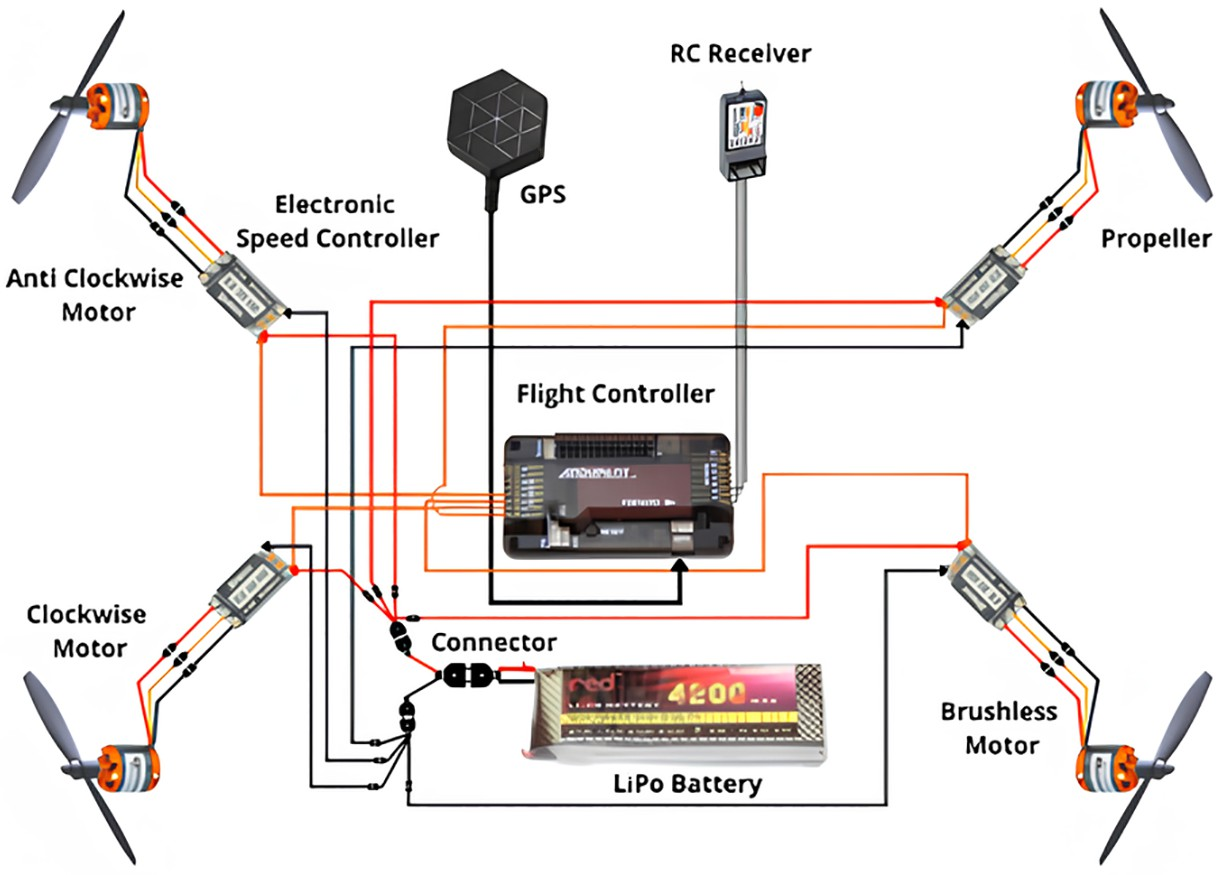
Schematic diagram of the quadcopter.

The components used to build the quadcopter are listed in the table (Table 1) below. The schematic of our quadcopter is given in the following diagram (Fig 5).

**Table 1:** List of components used for the construction of UAV quadcopter.

Name	Model	Quantity
Frame	YoungRC XL8 360mm FPV Racing Drone Frame	1
Motor	Brushless DC motor	4
Electronic Speed Controller	N/A	1
Propeller	DJI 9-Inch Thrust Boosted Self-Tightening 9450 Propeller	4
Flight Controller	YoungRC APM-2.8 Flight Controller Board	1
Remote Control	N/A	1
Battery	Rechargeable AA Battery	8
Video Transmitter and Receiver	ImmersionRC Tramp HV 5.8 GHz Video TX FPV Transmitter	1
Camera	Camera Specs of the Mounted Smartphone	1

**Fig 6:**
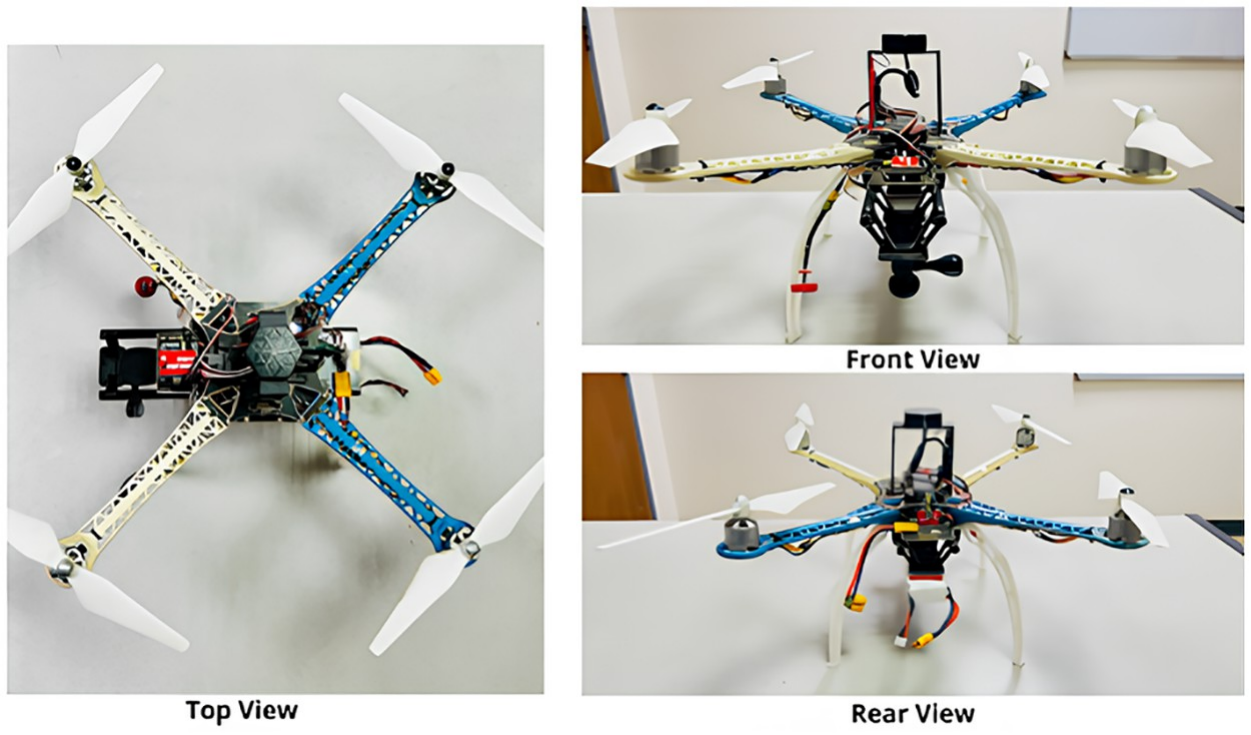
Images of the quadcopter from different angles.

Images of our quadcopter are given below (Fig 6).

### 3.7 UAV specifications

**Fig 7:**
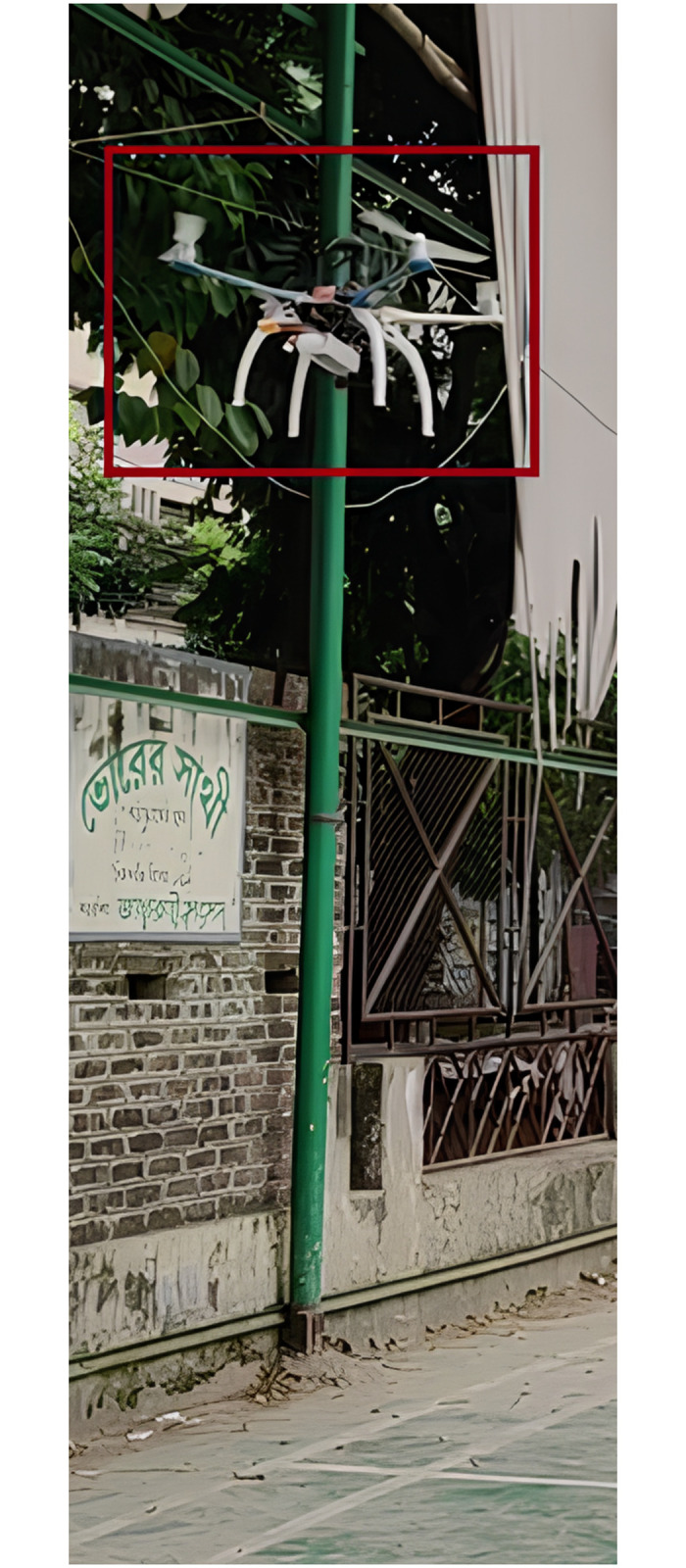
Drone in flight for data collection.

To get the finest freestyle recording, we chose the YoungRC XL8 360mm FPV Racing Drone Frame Carbon Fiber 8-inch FPV Freestyle Frame Kit with 4mm Arms. We have complete control over weight distribution because of its compact design. Additionally, the drone body is made entirely of 3K carbon fiber, which makes it sturdy but lightweight. It is also simple to assemble, making it ideal for FPV racing drones. A snapshot of the drone in-flight is provided in the next figure (Fig 7).

The 2312 motor adopts a pioneering stator winding structure, which not only makes it tidy but also increases slot wire embedding degree and offers improved heat dissipation. With its impact-resistant bearings, the system is more durable than ever before. The electromagnetic design has effectively improved the output power. The motor has a recommended Take-off Weight: 300g/axis and working temperature: -5°C 40°C. The stator size is 2.3 x 1.2 cm and weighs up to 55g.

The electronic speed controller, also known as an ESC, has the following dimensions: (3.2 x 1.6 x 0.5) cm, a current rating of 35A (40A burst), and a voltage rating that ranges from 3S to 6S. It can weigh up to 4.73g and 8.1g (PCB only) (with wires). The built-in sensor can be programmed for protection and can react to changes in temperature, output power, output current, etc. It features an integrated programmable RGB LED, a 32-bit ARM Cortex MCU STM32F051 48MHZ CPU, a throttled 2048 resolution ratio, and high interference resistance. The built-in sensor can be programmed for protection and can react to changes in temperature, output power, output current, etc. It supports BL Heli 32 firmware, which can be updated or the configuration altered online using the signal wire.

The remote control we used has a highly reliable receiver sensitivity, uses less power and offers greater interference protection. Thanks to the bidirectional communication capacity of each transmitter, it can receive data from temperature, altitude, and many other kinds of sensors, as well as from servo calibration and i-BUS support. There is a unique ID for each transmitter and receiver. The transmitter and receiver will only communicate with one another after being linked. This avoids inadvertent connections from other systems or interference with how the system operates. A strong, consistent connection is maintained while using less power and a high-efficiency omnidirectional high gain antenna, which also effectively eliminates interference. Despite utilizing extremely sensitive, low-power components, the system maintains great receiver sensitivity.

We used a Li-Polymer battery with a 6S cell count and a 4000 mAh capacity. It can withstand 22.2 V of electricity. It has the following measurements: 13.6 x 4.2 x 4.8 cm; weight: up to 596 g. Its large battery capacity guarantees a longer lifespan.

## 4 Data collection

The data for our study was collected using a UAV at Road#1,Block:D,Banasree,Dhaka-1219 location with permission from the concerned local authorities. The relevant document(s) have been provided as attachments.

## 5 Detection model

**Fig 8:**
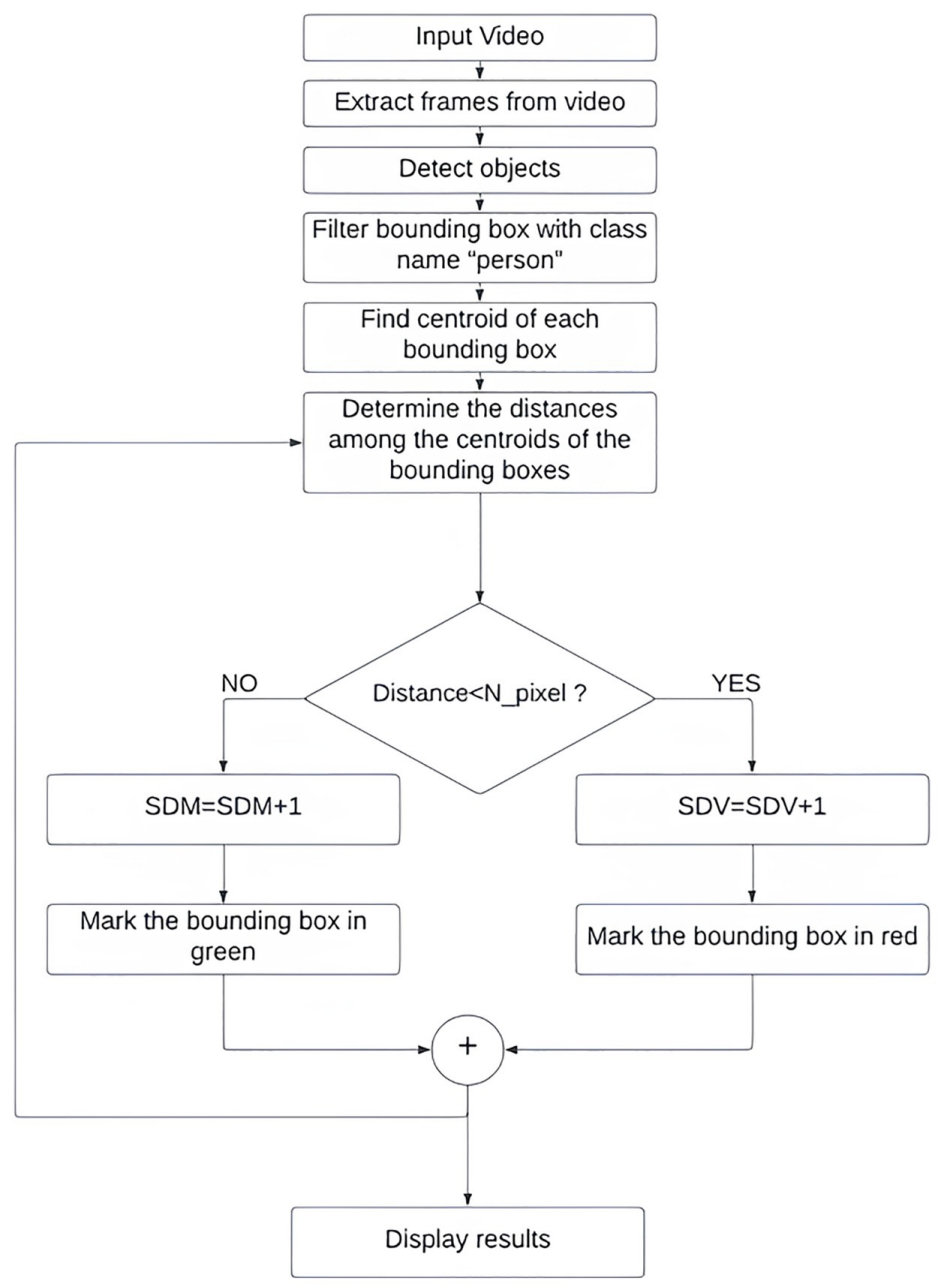
Flowchart for our proposed model. Here, SDV = Number of Social Distance Violations, SDM = Number of Social Distance Maintained.

By measuring the approximate distances between the ROIs, the software detection component has been utilized to recognize human objects, or the Region of Interest (ROI), from the split video frames and to detect violations of social distancing laws. The following flowchart provides a concise representation of the overall detection paradigm for the social distance violation (Fig 8). The overall computational complexity of our algorithm shown in the flowchart is O(n^2^).

### 5.1 ROI identification

A cutting-edge object recognition technique known as YOLO (You Only Look Once) is incredibly effective and well-known for detecting things with high accuracy in real time. A single neural network is used to process the entire image to accomplish quick object detection in real time. Bounding boxes with weighted probabilities are then created around prospective Sections of Interest to partition the image into distinct regions for object detection (ROIs). YOLO uses a multilabel classifier, making it possible to anticipate the class labels of the detected objects as well as their positions. There are numerous variations of the YOLO concept. For more accurate and effective ROI detection in our work, we preferred YOLOv4.

#### 5.1.1 YOLOv3

**Fig 9:**
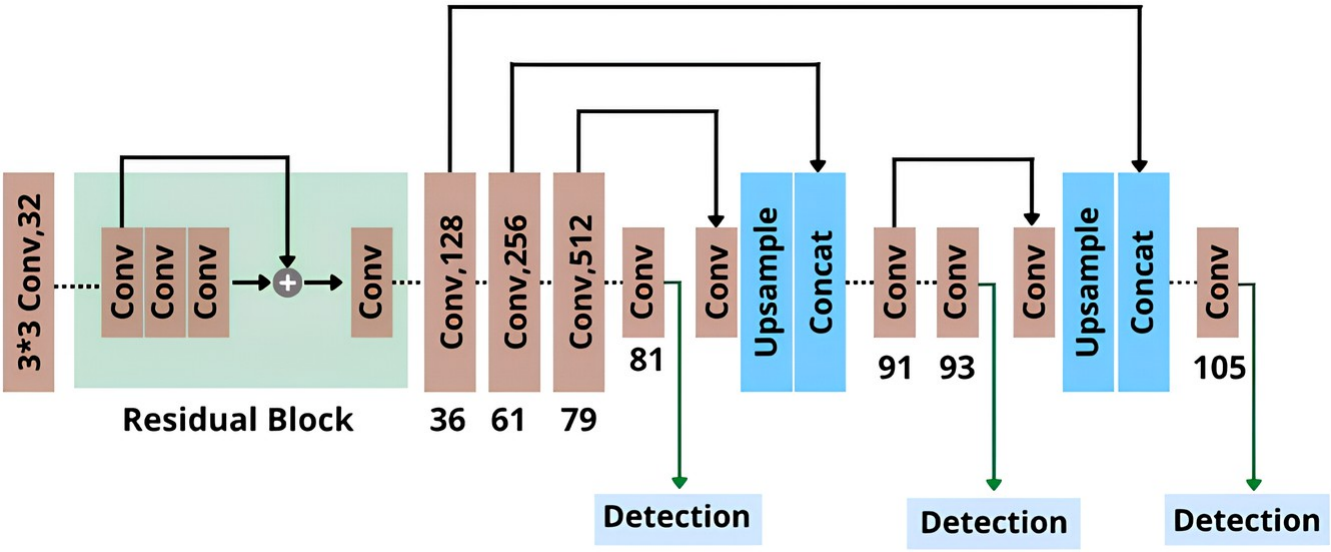
Model architecture of YOLOv3.

YOLOv3 is a much upgraded and more sophisticated version of YOLO that increases accuracy and speed using DarkNet53 as a backbone. It uses Convolutional Neural Networks (CNN) as an object-detection system in real-time to construct bounding boxes around objects, then predicts a probabilistic score for each bounding box using logistic regression for class identification [24]. This algorithm uses independent logistic classifiers to do multi-label classification. Additionally, the loss function for class predictions is binary cross-entropy loss. When overlapping labels are present in complicated datasets, the multi-label technique yields better results. A block diagram of the YOLOv3 architecture is given below [25] (Fig 9).

#### 5.1.2 YOLOv4

A more efficient version of YOLO known as YOLOv4 can operate quite successfully with just one GPU and a reduced mini-batch size. With YOLOv4, an incredibly quick and precise object detector can be trained on a single 1080 Ti or 2080 Ti GPU, making it more widespread in terms of GPU utilization than YOLOv3. When used with a Tesla V100 and running at 65 FPS, YOLOv4 achieves 43.5% AP on the MS COCO dataset when combined with Cross mini-Batch Normalization (CmBN), Weighted Residual Connections (WRC), Cross Stage Partial connections (CSP), Self-Adversarial Training (SAT), Mosaic Data Augmentation, Drop Block Regularization, Mish Activation, and CloU loss [26]. After 16474 iterations on the MS COCO dataset with a resolution of 608, YOLOv4 obtained an AP score of 74% [27]. The extra building blocks found in YOLOv4 are referred regarded as universal features since they work with any computer vision workloads, datasets, and models. These features enhance YOLOv4’s portability and effectiveness on multiple datasets. Additionally, YOLOv4 incorporates cutting-edge methods like “Bag-of-Freebies” and “Bag-of-Specials” to greatly increase item detection accuracy. The performance of YOLOv4 on the MS COCO dataset is contrasted with that of various object detection models in the table below (Table 2) [28].

**Table 2:** Performance comparison of YOLOv4 model and the other existing models on MS-COCO dataset [28].

Model	AP_S_	AP_M_	AP_L_	FPS
SSD	6.14	24.85	40.52	25.2
YOLOv3	10.62	29.36	42.17	32.5
YOLOv4	19.72	42.58	55.63	30.6
YOLOv3-SPP	19.52	35.13	45.52	27.5

**Fig 10:**
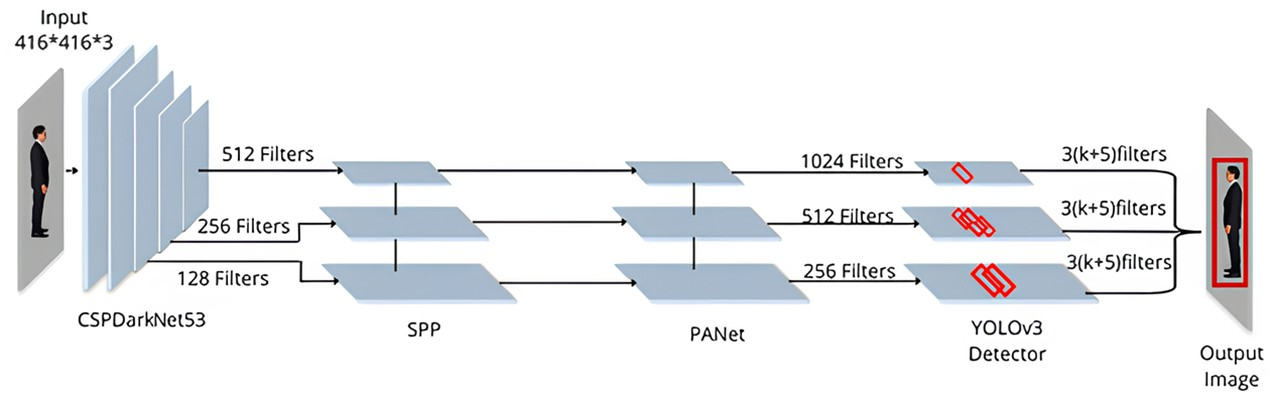
Model architecture of YOLOv4.

Our model is divided into three main components: the head, neck, and backbone. A neural network that was trained on the picture categorization from the MS COCO dataset serves as the framework. Between the backbone and the head, there are a few extra layers where feature maps from various stages are collected. The head, the third component, is utilized to generate bounding boxes for objects and predict classes. Here, CSPDarkNet-53 serves as the framework for both object detection and receptive field expansion [29]. This will ensure that significant contexts can be extracted from the image. To keep the network operation speed constant, SPP has been used. Furthermore, PANet was used as neck to aggregate the parameters from different CSPDarkNet-53 levels. This neural network architecture has 53 convolutional layers where the network is built with consecutive 3x3 and 1x1 convolution layers. The 53 layers of the DarkNet are stacked with 53 more layers for the detection head which results in a total of 106 layers of fully convolutional underlying architecture. The number of filters starts with 32 and is doubled at every layer and a residual group Spatial Pyramid Pooling (SPP) layer is added to remove the fixed-size constraint of the network. This layer is added between convolutional layers and fully connected layers to avoid the need for cropping at the beginning. This model was then integrated with YOLOv3 to complete our design (Fig 10).

### 5.2 Coordinate estimation of ROI

In YOLO, prediction of the bounding box of a detected object requires 4 coordinates namely *t*_*x*_, *t*_*y*_, *t*_*w*_, *t*_*h*_ where *x* and *y* represent the coordinate axes and *w* and *h* stand for width and height respectively. If the cell is shifted from the top left corner of the image by (*c*_*x*_, *c*_*y*_) and the bounding box has prior width and height of *p*_*w*_, *p*_*h*_ respectively then the predictions will be given by the following equations.

$$\begin{array}{c} b_{x}=\sigma \left(t_{x}\right)+c_{x} \end{array}$$ (40)

$$\begin{array}{c} b_{y}=\sigma \left(t_{y}\right)+c_{y} \end{array}$$ (41)

$$\begin{array}{c} b_{w}=p_{w}\;\text{exp}\;\left(t_{w}\right) \end{array}$$ (42)

$$\begin{array}{c} b_{h}=p_{h}\;\text{exp}\;\left(t_{h}\right) \end{array}$$ (43)

$$\begin{array}{c} \text{Prob}(\text{object})\times \text{IOU}\left(b,\text{object}\right)=\sigma \left(t_{0}\right) \end{array}$$ (44)

**Fig 11:**
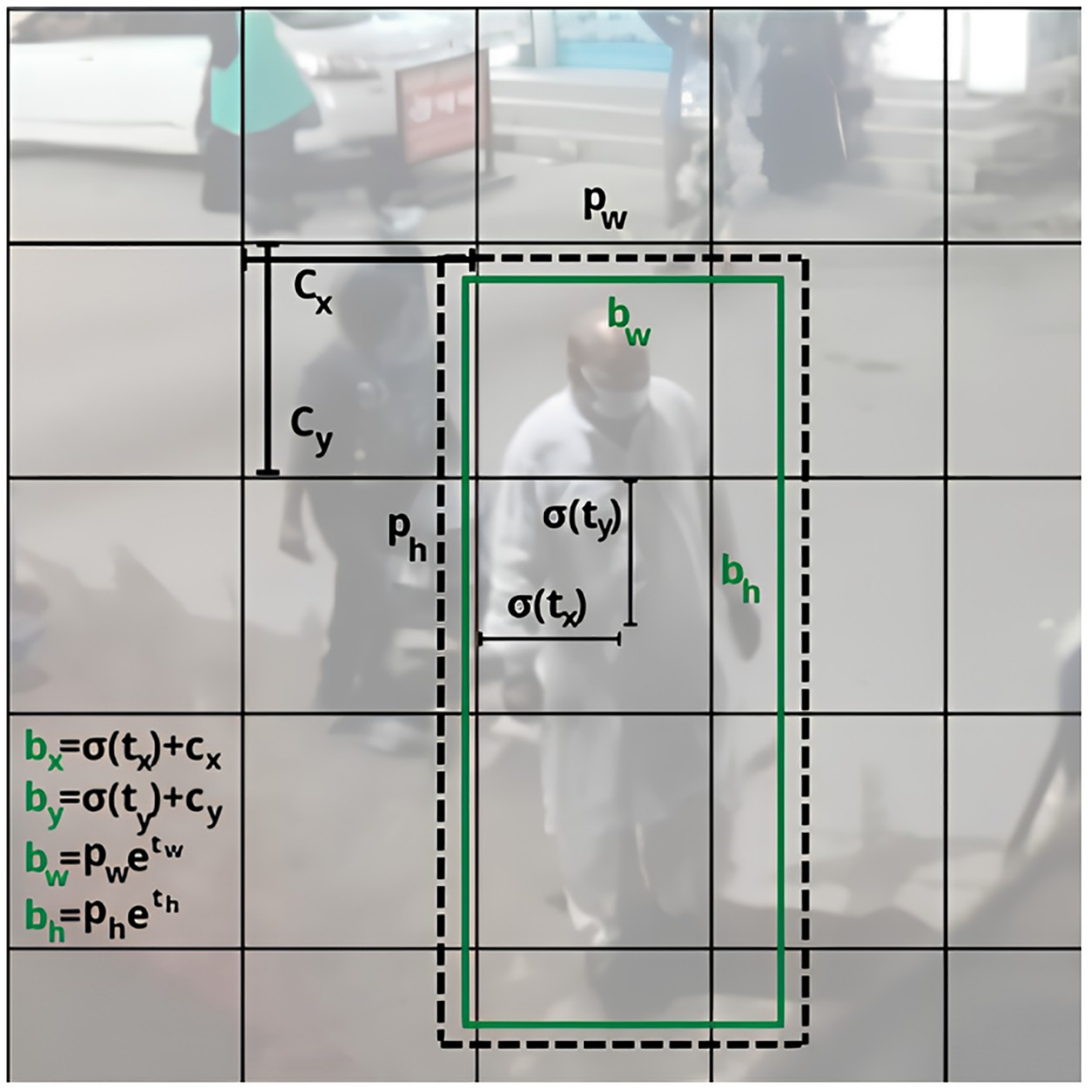
Bounding box with location priors and location prediction.

**Fig 12:**
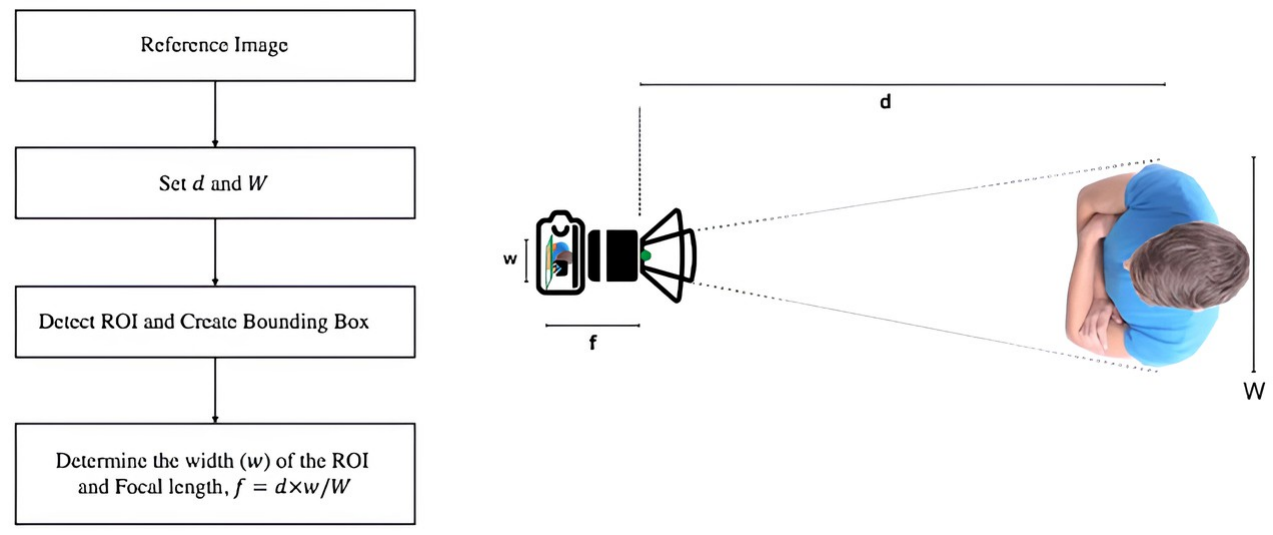
Process for determining the focal length (*f*) of the camera.

The bounding box is demonstrated in the following figure (Fig 11). We must translate the location of the bounding boxes into corresponding coordinates to ascertain the positions of the individuals in the video. The rectangular coordinate system was employed for this. The center coordinate of the bounding boxes can be found using our object detection model. The YOLOv4 component of our model draws bounding boxes around matching items, such as people moving about in the street. The center of each bounding box can be located using three coordinates, namely *x*, *y*, and *d*, where *d* is the separation between the detected item and the camera lens. Our model was trained with objects that are positioned at a preset distance from the camera to calculate *d*. find the items’ distance from the camera. The camera’s focal length, *f*, was calculated using a reference image to fine-tune the model. The following equations can be used to calculate the separation of objects from the camera once the model has been appropriately tuned (Fig 12).

$$\begin{array}{c} f=\frac{wd}{W}\Rightarrow d=\frac{Wf}{w} \end{array}$$ (45)

Here, *f* = focal length of the camera (scaled to pixels of the camera), *d* = distance (in meters) of the detected object from the camera, *w* = width of the bounding box surrounding the detected object (scaled to pixels of the camera), W = approximate average width of the detected object.

For a group containing *N* number of objects, we can use the following equation.

$$\begin{array}{c} D=[\begin{array}{cccc} f & 0 & \cdots & 0\\ 0 & f & \cdots & 0\\ \vdots & \vdots & \ddots & \vdots \\ 0 & 0 & \cdots & f \end{array}][\begin{array}{c} W/w_{1}\\ W/w_{2}\\ \vdots \\ W/w_{N} \end{array}] \end{array}$$ (46)

**Fig 13:**
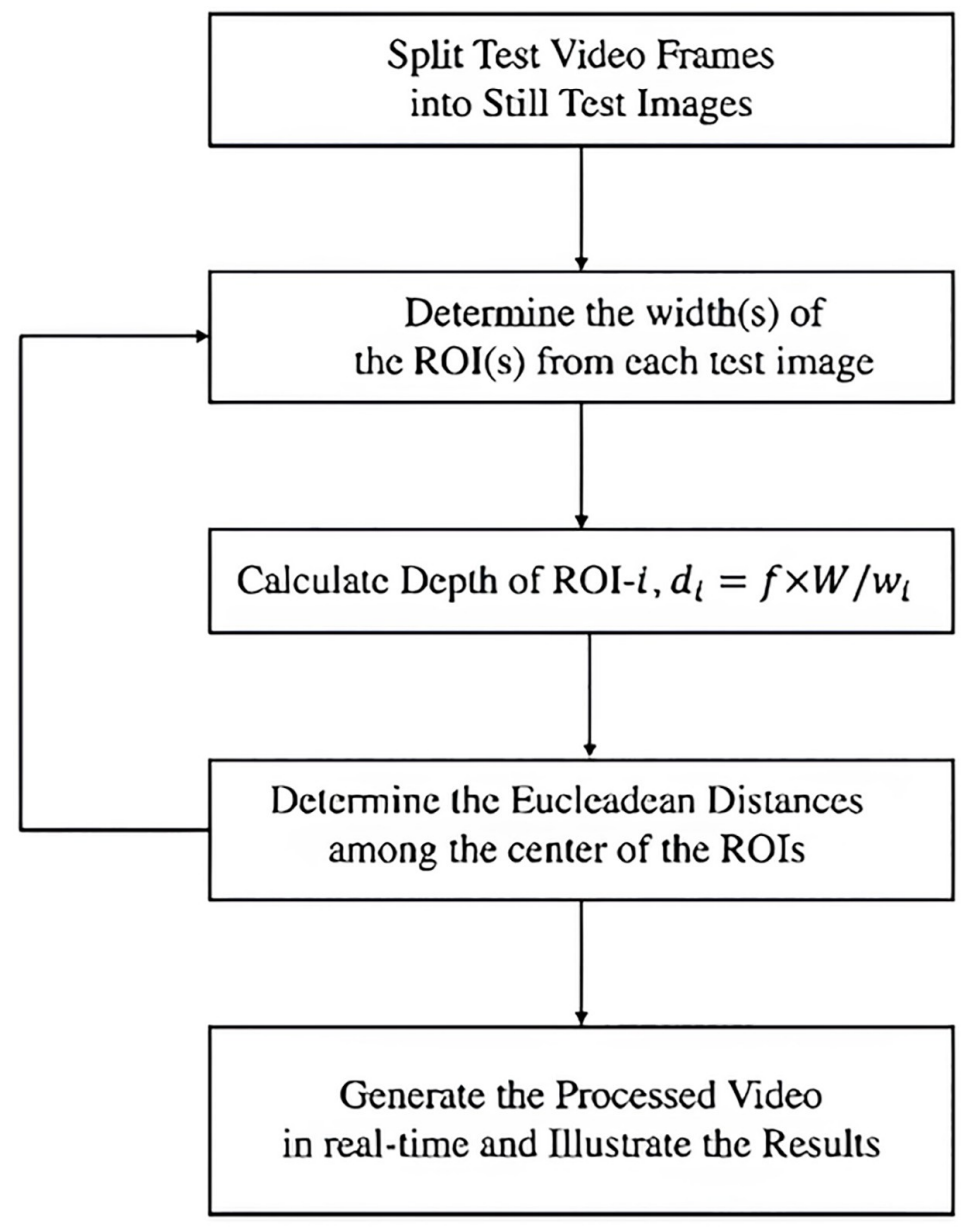
Process of estimating distance (*d*) of ROIs from the camera.

Note that, in our model, the approximate width of a person was assumed to be 0.411 meter [30]. The process of estimating the distance of ROIs from from the camera is demonstrated in the following figure (Fig 13).

We have used Euclidean distance as the method for estimating the distances among the ROIs. In the three-dimensional Euclidean space, the distance between *p* = (*p*_1_, *p*_2_, *p*_3_) and *q* = (*q*_1_, *q*_2_, *q*_3_) is given by the following equation.

$$\begin{array}{c} d\left(p,q\right)=\sqrt{{\left(q_{1}-p_{1}\right)}^{2}+{\left(q_{2}-p_{2}\right)}^{2}+{\left(q_{3}-p_{3}\right)}^{2}} \end{array}$$ (47)

Once the coordinates of individuals have been determined, the distances among them can be estimated using the formulae discussed above.

## 6 Result & discussion

Our study used YOLOV4 real-time analysis on video that was recorded by a camera-mounted UAV to tally the instances of social distance violations. Using appropriate geometrical analysis that included determining the distances of those ROIs from the source, the approach involved estimating the distances between the discovered ROIs. We used UAV to produce our data as many real-time videos for analysis. The videos had an average runtime of 30 seconds at a frame rate of 25 fps, containing roughly 750 frames. These frames were then divided into still images and fed into the YOLOv4 model that had already been trained. The corresponding results are analyzed thoroughly in the following sections.

### 6.1 Model calibration, detection, and depth calculation

**Fig 14:**
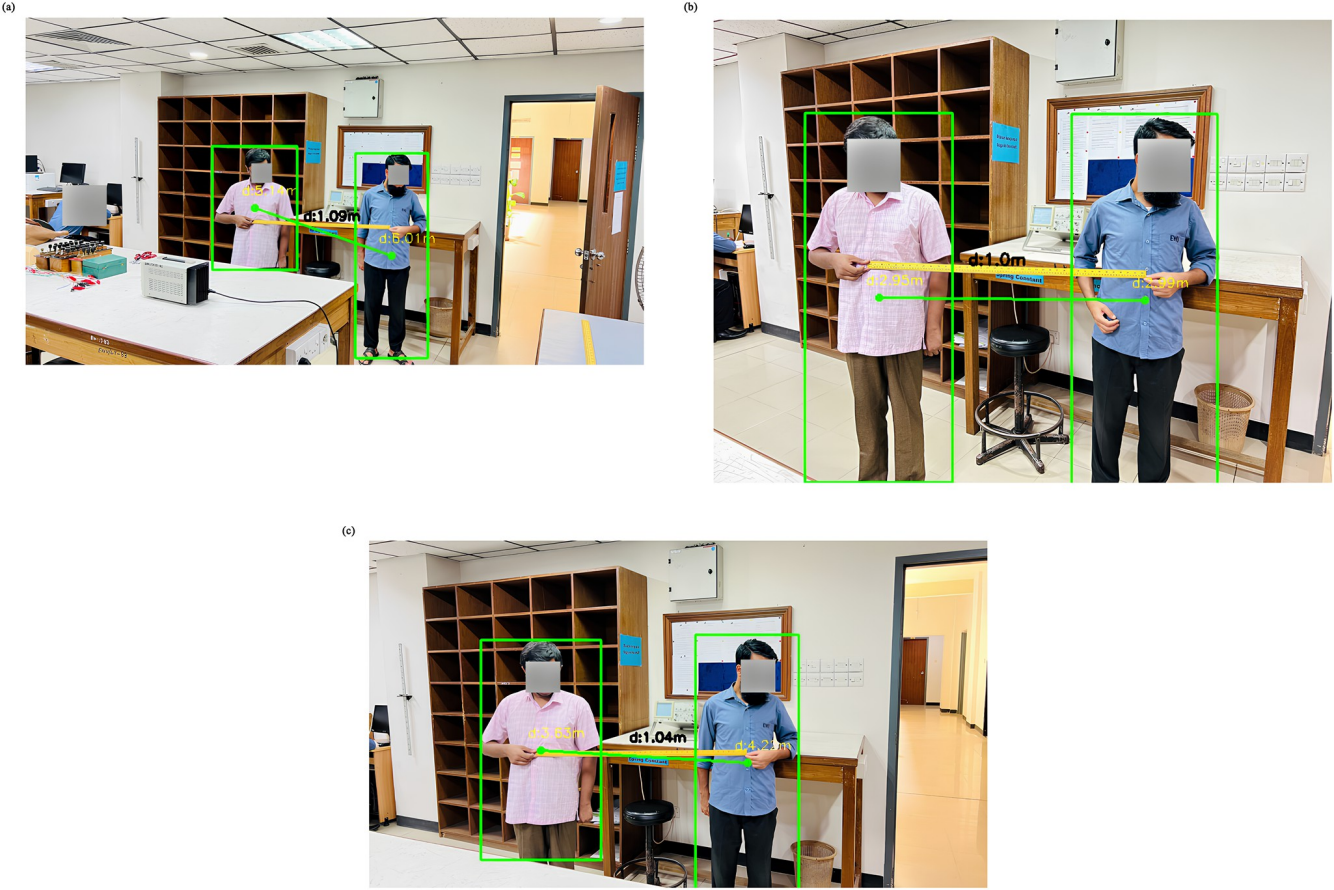
(a–c) Benchmarking the distance calibration (Courtesy to the physics laboratory of East West University).

Significant parameters used in our study are confidence, non-maximum suppression threshold, minimum distance in pixel, known distance, and known width. To enhance performance under a relatively low light environment, the confidence value was chosen to be 0.3 on a scale of 1. The non-maximum suppression threshold was set to 0.4 to ensure higher detection with a higher success rate. We calibrated the known distance with different angles and different distances and used the known distance of the reference image as 1.2 meters which will be compared with our input videos and return the approximate distance from the source. We must calibrate the camera lens in order to measure human distances more precisely. We have given our network a few still images of two people with a known distance of 1 meter (which has been preserved using a meter scale) taken from various postures and angles with respect to the lens to calibrate the camera lens for appropriately measuring the social distances (Fig 14a–14c).

**Fig 15:**
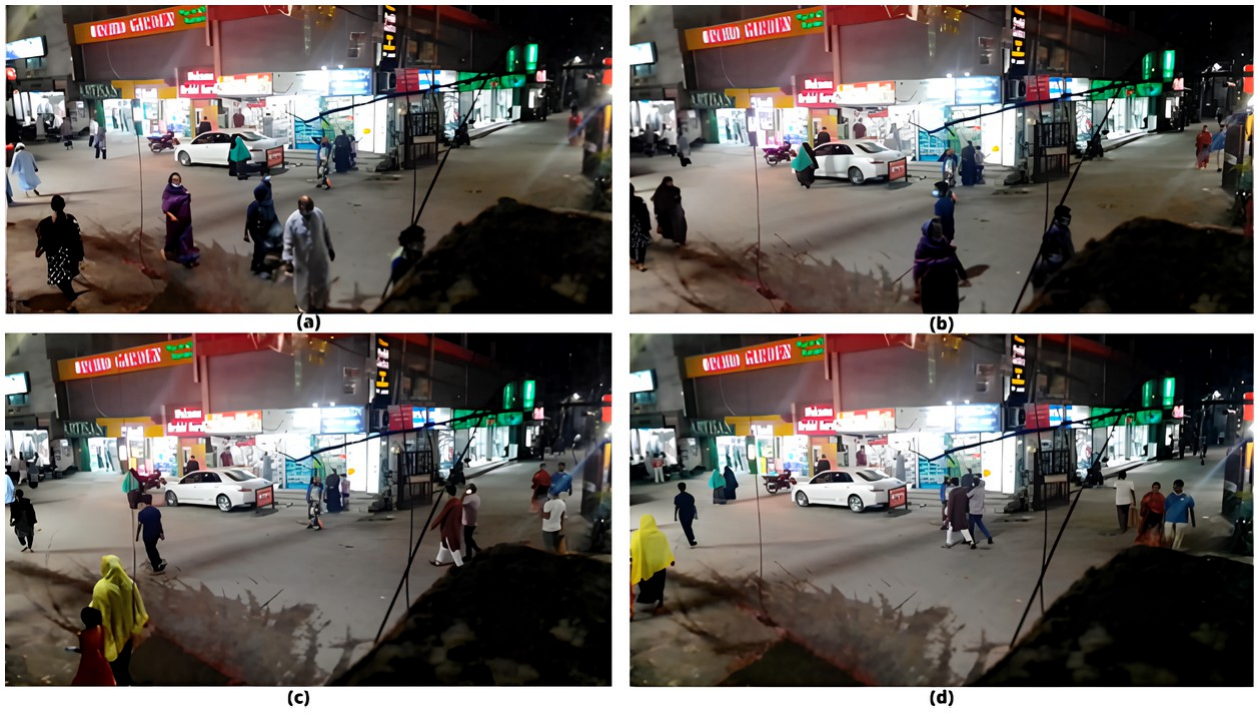
Raw frames before object detection (a—d).

CSPDarkNet53 served as the foundation of YOLOv4’s object detection system. Fig 15 displays some sample real-time video raw images that were gathered. These pictures served as the object detection input. To acquire the ROIs, CSPDarkNet53 classified the identified items. Higher ROI detection accuracy was obtained as a result of using SPP and PANet as additional layers in the neck of our model.

**Fig 16:**
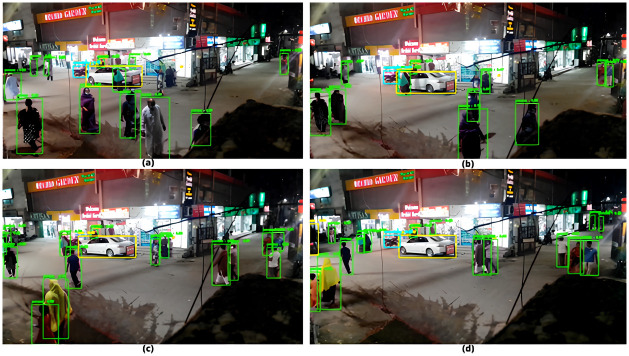
Processed frames with detected ROIs (a—d).

The results obtained from the above-mentioned frames are shown in Fig 16 below.

As evident from Fig 16 above, the object detector performed expectedly to detect ROIs. In Fig 16(a), the accuracy of detection of ROI was 80% whereas in Fig 16(c) the accuracy turned out to be 100%. A similar level of accuracy was observed for other frames too.

**Fig 17:**
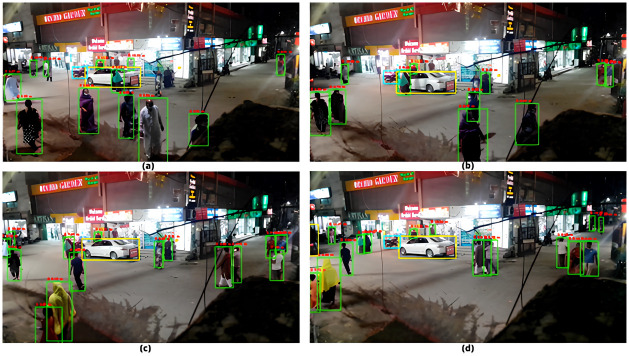
Images after depth calculation (a—d).

Fig 17 demonstrates the output frames obtained after estimation of the depths of the ROIs from the camera lens. Our model, which was calibrated to estimate the distance from the pixel width of ROI, successfully estimated the distances of the ROIs from the camera as can be seen from the frames in the figure given above.

**Fig 18:**
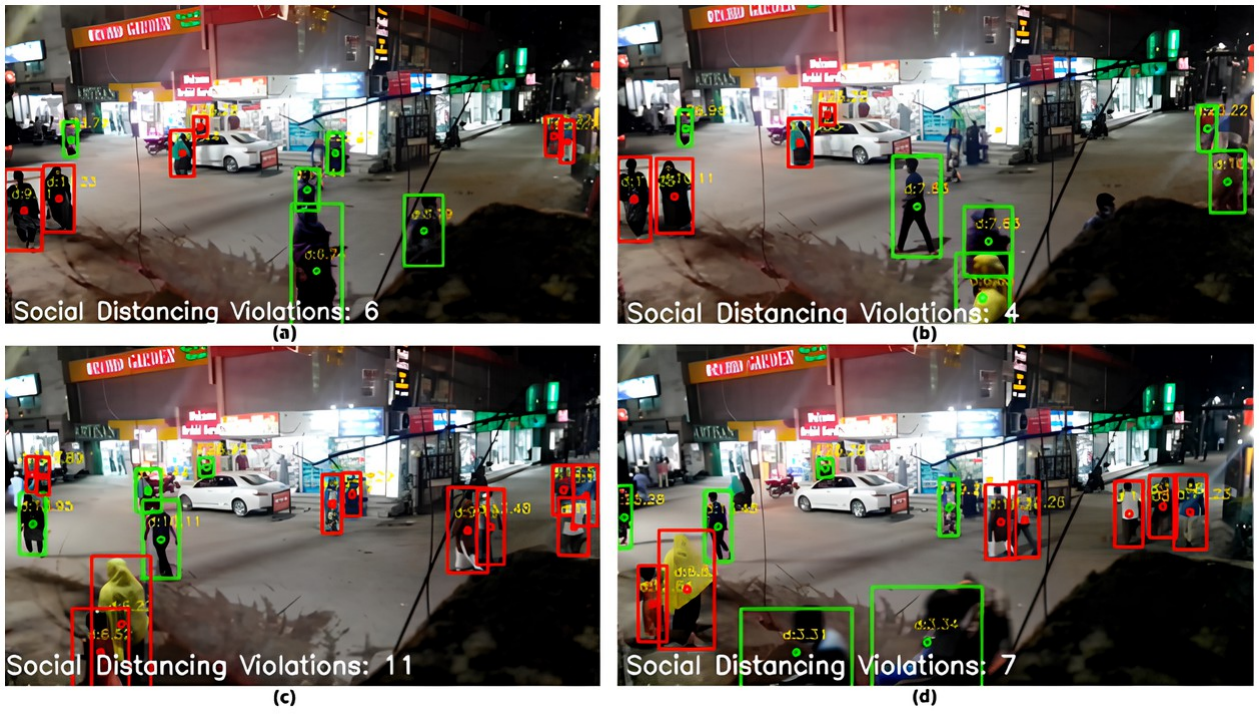
Processed images displaying the count of social distance violations (a—d).

The Euclidean distances between ROIs were calculated after the coordinates of the ROI centers were established. As shown in Fig 18, the respective ROIs were indicated with red bounding boxes if the distances were less than the social distancing rule’s accepted minimum distance. As a result, the number of such breaches was quickly counted and displayed.

### 6.2 Performance evaluation

**Fig 19:**
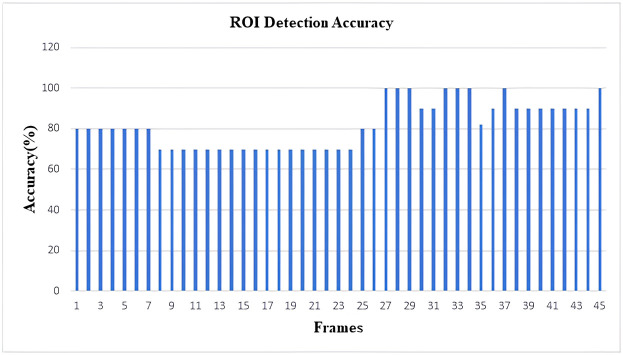
Histogram of accuracy for detecting ROIs (From Video-1).

**Fig 20:**
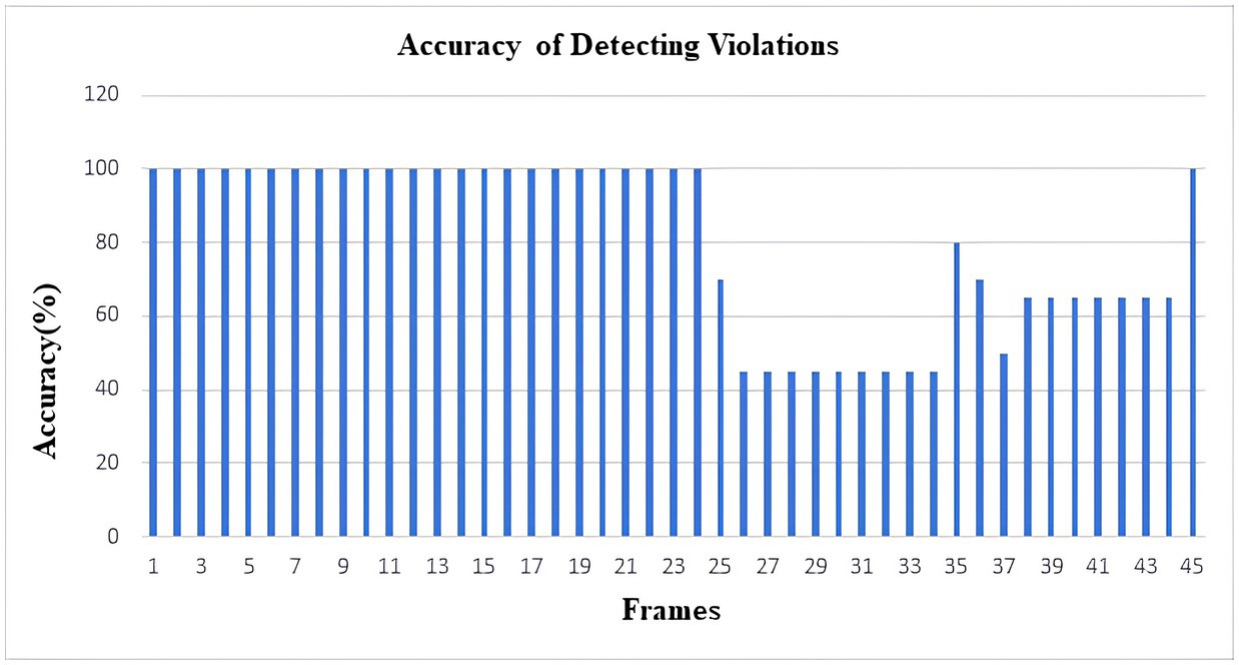
Histogram of accuracy for detecting social distance violations (From Video-1).

**Fig 21:**
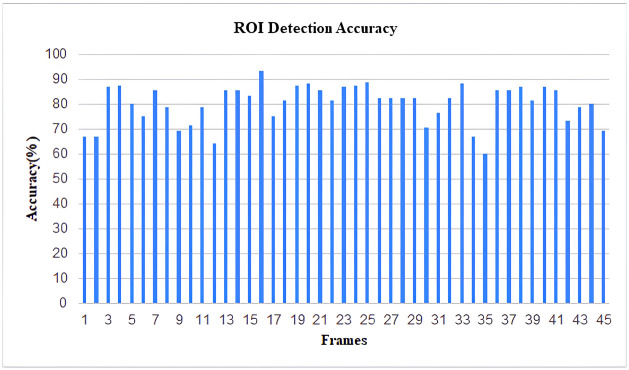
Histogram of accuracy for detecting ROIs (From Video-2).

**Fig 22:**
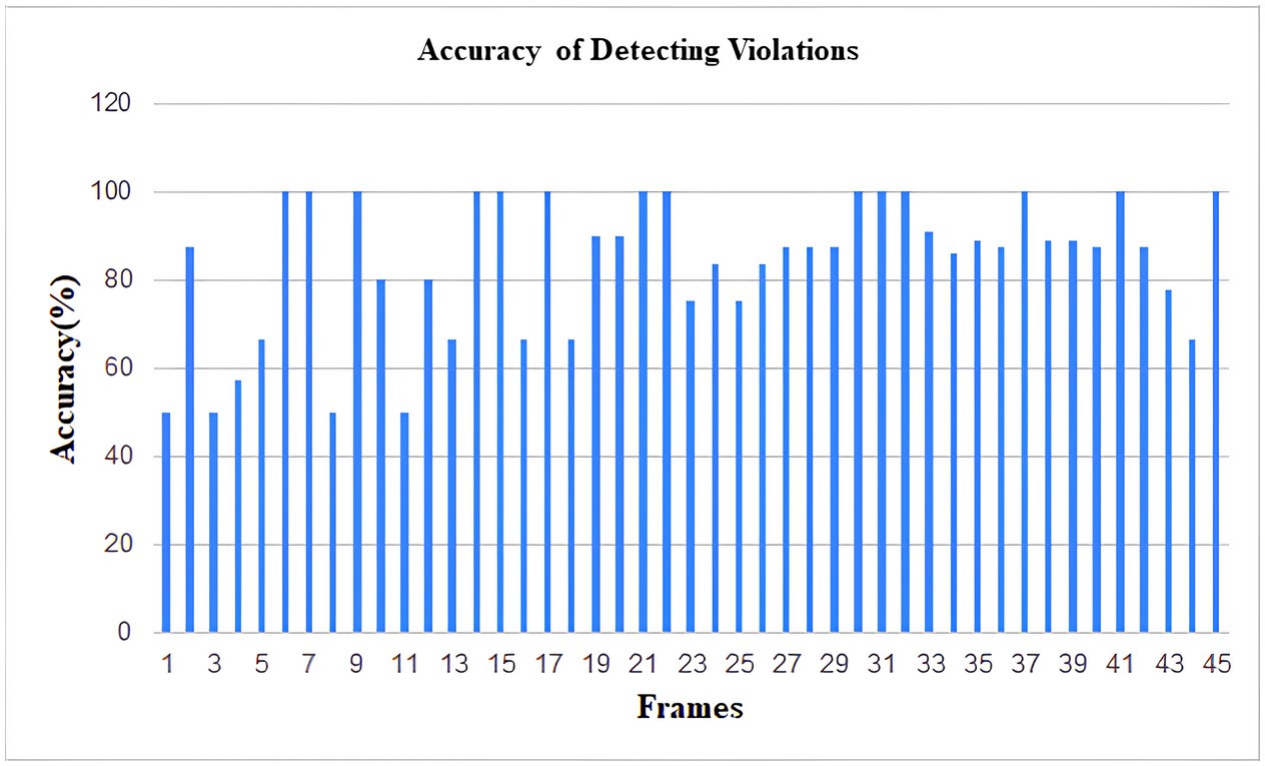
Histogram of accuracy for detecting social distance violations (From Video-2).

As YOLOv4 uses CSPDarkNet53 as the backbone of the network architecture, this neural network provides superior performance in terms of detecting objects on the MS COCO dataset. As a result, the inclusion of YOLOV4 in our study aimed at the detection of violations of social policies produced efficient and adequately accurate results. As our work involves detecting items of a wide variety of sizes, the usage of the SPP block in CSPDarkNet53 considerably enhances the receptive field, allowing the model to detect many objects of varied sizes. The predicted accuracy of the ROI identification has been found to be around 85% following the implementation of the YOLOv4 model on our dataset. Every frame produced by the video feed was taken into account while calculating the detection accuracy of the model. Additionally, using a similar method to that described above, the expected accuracy of recognizing the number of social distance violations was calculated, and it came out to be around 82%. The ensuing figures illustrate the outcomes. The results are demonstrated in the following figures (Figs 19–22).

### 6.3 Future prospects

During the COVID-19 pandemic, we intended to employ a unique method for detecting social distance violations. Nonetheless, our concept can be applied to any circumstance where social separation is necessary. Despite utilizing an existing object detection algorithm for our model, our methodology distinguishes our work as innovative within the relevant study field. Although the scope of our study was limited to the detection of social distance violations, we intend to expand it in the future for more general applications, such as the observation of behaviors among various gender and age groups in relation to social distance or any other public health-related restrictions. The data gathered by our model that has been enhanced with additional parameters can be used to build datasets for subsequent real-time analyses of the same kind. Even though our model produced satisfactory results, it might be prone to camera quality issues in bad weather. The use of a higher-resolution camera can increase the reliability of our model.

## 7 Conclusion

The COVID-19 pandemic has dramatically affected the way humans live. To protect the public’s health amid the epidemic, innovative technology was required. Maintaining social distance among people is critical because the disease is contagious. Our research focused on the application of cutting-edge technologies to ensure that social distancing is carried out correctly. Our system has proven to be quite effective with our model in identifying social distance violations with a high degree of accuracy (82%) on the real-time dataset, which is quite encouraging compared to similar contemporary efforts. With the aforementioned architecture, our system can be installed on any practical equipment suitable for monitoring COVID procedures in large public places. As part of the research, we may adapt and apply our model to identify face masks and other COVID measures in real-time practical scenarios, hence raising public awareness of health and safety concerns associated with such pandemics in the future.
